# Clinical relevance of somatic mutations in Chinese lung adenocarcinoma and their prognostic implications for survival

**DOI:** 10.1002/cam4.7227

**Published:** 2024-05-21

**Authors:** Tongxin Li, Jie Liu, Yu Zhou, Shengyuan Huang, Dong Wang, Jianrong Chen, Yong Fu, Ping He

**Affiliations:** ^1^ Department of Cardiothoracic Surgery Dianjiang People's Hospital of Chongqing Chongqing China; ^2^ Department of Thoracic Surgery, Southwest Hospital Army Medical University (Third Military Medical University) Chongqing China; ^3^ Department of Cardiac Surgery, Southwest Hospital Army Medical University (Third Military Medical University) Chongqing China

**Keywords:** clinical characteristic‐related genes, lung adenocarcinoma, MPGM risk model, mutation profiles, targeted sequencing

## Abstract

**Background:**

To comprehensively elucidate the genomic and mutational features of lung cancer cases, and lung adenocarcinoma (LUAD), it is imperative to conduct ongoing investigations into the genomic landscape. In this study, we aim to analyze the somatic mutation profile and assessed the significance of these informative genes utilizing a retrospective LUAD cohort.

**Methods:**

A total of 247 Chinese samples were analyzed to exhibit the tumor somatic genomic alterations in patients with LUAD. The Cox regression analysis was employed to identify prognosis‐related genes and establish a predictive model for stratifying patients with LUAD.

**Results:**

In the Dianjiang People's Hospital (DPH) cohort, the top five frequent mutated genes were (Epidermal growth factor receptor) EGFR (68%), TP53 (30%), RBM10 (13%), LRP1B (9%), and KRAS (9%). Of which, EGFR is a mostly altered driver gene, and most mutation sites are located in tyrosine kinase regions. Oncogene pathway alteration and mutation signature analysis demonstrated the RTK‐RAS pathway alteration, and smoking was the main carcinogenic factor of the DPH cohort. Furthermore, we identified 34 driver genes in the DPH cohort, including EGFR (68%), TP53 (30.4%), RBM10 (12.6%), KRAS (8.5%), LRP1B (8.5%), and so on, and 45 Clinical Characteristic‐Related Genes (CCRGs) were found to closely related to the clinical high‐risk factors. We developed a Multiple Parameter Gene Mutation (MPGM) risk model by integrating critical genes and oncogenic pathway alterations in LUAD patients from the DPH cohort. Based on publicly available LUAD datasets, we identified five genes, including BRCA2, Anaplastic lymphoma kinase (ALK), BRAF, EGFR, and Platelet‐Derived Growth Factor Receptor Alpha (PDGFRA), according to the multivariable Cox regression analysis. The MPGM‐low group showed significantly better overall survival (OS) compared to the MPGM‐high group (*p* < 0.0001, area under the curve (AUC) = 0.754). The robust performance was validated in 55 LUAD patients from the DPH cohort and another LUAD dataset. Immune characteristics analysis revealed a higher proportion of primarily DCs and mononuclear cells in the MPGM‐low risk group, while the MPGM‐high risk group showed lower immune cells and higher tumor cell infiltration.

**Conclusion:**

This study provides a comprehensive genomic landscape of Chinese LUAD patients and develops an MPGM risk model for LUAD prognosis stratification. Further follow‐up will be performed for the patients in the DPH cohort consistently to explore the resistance and prognosis genetic features.

## Graphical abstract

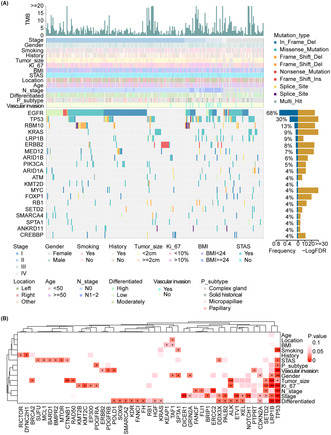

## 1 INTRODUCTION

As is reported in Global cancer data, lung cancer is the leading cause of cancer‐related death.^1^ Based on the histological classification, non‐small cell lung cancer (NSCLC) accounts for approximately 85% of lung cancer cases, and lung adenocarcinoma (LUAD) is the most prevalent subtype of NSCLC.^2^

Multiple studies and expert consensus have furnished compelling evidence linking the survival of cancer patients to clinical and pathological characteristics, including but not limited to age, clinical stage, smoking status, and family history, among others.^3^ Tu et al. prospectively studied the association of smoking characteristics with all‐cause mortality in 23 cancer types. They proved the significant harms of current smoking on survival after a cancer diagnosis. In lung cancer, current smokers and former smokers were at greater risk of death.^4^, ^5^ Guerreiro et al. proposed that with the increasing age (>50) and clinical stage at diagnosis, relative survival decreased progressively based on a large population study.^5^, ^6^ In addition, tumor spread through airspaces (STAS) and vascular invasion is aggressive clinical pathology characteristics, independent poor prognosis factors for recurrence‐free survival in NSCLC according to the multivariate Cox regression analysis.^7^, ^8^ Abundant clinical features and prognostic biomarkers are critical in guiding precise therapy decisions and improving survival outcomes. However, most studies primarily focus on the prognostic significance of these clinical features, with limited evidence regarding the association between somatic tumor alterations and clinicopathologic features. Therefore, there is an urgent need for robust genomic evidence to unravel the molecular mechanisms linking gene alterations to clinicopathologic characteristics in LUADs.

With continual advancement and iteration of next‐generation sequencing (NGS) technology, abundant driver gene mutations are identified, leading to a more comprehensive genomic landscape of LUAD.^9^ Several studies have verified that tumor genome alterations are closely related to cancer occurrence and development. In addition, driver gene and oncogenic pathway alterations could also provide effective targeted therapy opportunities.^10^ The receptor tyrosine kinase (RTK)/RAS/RAF pathway genes are frequently mutated in LUAD.^11^ Recurrently, somatic genomic alterations in genes such as EGFR, KRAS, BRAF, and others have emerged as conventional therapeutic targets, leading to revolutionary improvements in clinical outcomes.^12^

Nevertheless, targeted therapy's vulnerability, characterized by the emergence of recurrent drug resistance and adverse reactions, highlights the imperative to gain a deeper understanding of tumor‐specific somatic alterations and the underlying molecular mechanisms in LUAD. Despite the significant advancements in clinical trials for targeted therapy and immunotherapy, which have improved clinical outcomes for patients with LUAD, the practical implementation of these effective treatment options remains restricted to a minority of patients. Notably, the 5‐year survival rate for patients with advanced clinical stages (III‐IV) remains below 60%.^1^, ^13^ Significant heterogeneity was observed among LUAD patients in histological subtypes and in genomic and cellular characteristics.^14^ Therefore, we aim to develop a prognosis risk model based on mutation characteristics to enable risk stratification for patients, mainly when treatment options are restricted.

In this study, we comprehensively analyzed genomic mutation characteristics in a Chinese LUAD cohort. We investigated the genes with significantly different mutations associated with clinical pathology risk factors, potentially influencing the development of specific clinical and pathological characteristics. Subsequently, a comprehensive screening was performed, incorporating these genes and hotspot mutations, driver gene alterations, and oncogenic pathway genes to identify a gene set that exhibits the strongest correlation with patient survival prognosis in LUAD. Leveraging these patient‐specific genomic alterations, we developed a predictive prediction model to assist clinicians in patient risk stratification. Our study provides new insight into the genomic alterations and prognosis stratification for LUAD.

## 2 MATERIALS AND METHODS

### 2.1 Data

The Dianjiang People's Hospital (DPH) cohort comprised 247 LUAD patients. The baseline information of patients in the DPH cohort was in Table S1, and the somatic mutation data were in Table S2.

The EAS cohort^15^ included 299 Chinese patients with LUAD and was used as a training cohort to develop the prognosis risk model. The corresponding clinical information and the TOP100 mutation genes were summarized in Tables S3 and S4, respectively. The RNA‐seq data of 169 patients with LUAD from the EAS cohort were also acquired from cBioPortal and published in the previous article.

The validation cohort (MSK‐LUAD)^16^ comprised 394 LUAD samples obtained from an Asian population within the MSK‐MET dataset (Memorial Sloan Kettering‐Metastatic Events and Tropisms, containing 3853), which was utilized to validate the predictive performance of the risk model. The mutation and corresponding clinical data of EAS and MSK‐LUAD cohorts were obtained from the cBioPortal (https://www.cbioportal.org/). The clinical information and the TOP100 mutation genes were comprehensively summarized in Tables S5 and S6, respectively.

Among 247 LUAD patients from the DPH cohort, 55 were treated with targeted or/chemotherapy /and immunotherapy and participated in regular follow‐up examinations. The detailed prognosis and therapy (drug type and adverse reaction) information are in Table S7. The last follow‐up was performed on 2nd June 2023, and progression‐free survival (PFS) was used as the survival endpoint.

### 2.2 Targeted sequencing

The formalin‐fixed paraffin‐embedded (FFPE) sample of the DPH cohort was profiled with targeting sequencing (1123 gene panel) (ChosenMed, Beijing, China), and germline variation was synchronously sequenced using the paired adjacent tissues or peripheral blood samples. DNA extraction from tumor specimens was performed using the Maxwell RSC FFPE Plus DNA Kit (Promega, Cat no.AS1720), followed by shearing of genomic DNA (gDNA) into approximately 200 bp fragments using a Covaris E210 system (Covaris, Inc.). Construction of the NGS library was accomplished using the KAPA HyperPrep Kit (Roche, 07962312001) and the Agilent SureSelect XT kit (Agilent, G9702C). The library's quantity was assessed with the Qubit 3.0 Fluorometer (Life Technologies, Inc.), while its quality and fragment size were determined using an Agilent 2100 Bioanalyzer (Agilent Technologies, Inc.). Finally, targeted sequencing of the gDNA was conducted, employing paired‐end sequencing on an Illumina NovaSeq 6000 platform (Illumina Inc) with a 150‐bp read length.

### 2.3 Data analysis

Somatic mutations were identified by aligning the human reference genome (UCSC hg19) using the Burrows‐Wheeler Aligner (BWA version 0.7.11) and subsequently calling mutations with the IndelRealigner tool from the Genome Analysis Toolkit (GATK, version 3.6)^17^ and VarScan software.^18^ Annotation was performed using ANNOVAR, and variants were filtered based on several criteria: exclusion of intronic variants, absence in more than 1% of the 1000 Genomes Project population, absence in dbSNP common Single Nucleotide Polymorphism (SNPs), exclusion of synonymous variants, removal of variants with <50 supporting reads, elimination of mutations flagged as “black flag,” and ensuring tumor cell content ≥10%, with an average sequencing depth ≥ 2000×, and DNA insert fragment size ≥140 bp.

### 2.4 Genomic alterations analysis

Tumor mutational burden (TMB) was determined by counting all nonsynonymous mutations and indels per megabase (MB) of genomic alterations. A threshold of 10 mutations per MB was used to classify tumors as either TMB‐high or TMB‐low. Driver genes of the EAS LUAD cohort were identified using MutSigCV^19^ method.

### 2.5 Development of the risk model

Multiple parameter gene mutations (MPGM) were screened with mutation frequency >0% in the training cohort, and the remaining candidate genes were further screened utilizing univariate and multivariable Cox regression analysis. The multivariable Cox regression was performed to develop the MPGM risk model, and the risk scores were calculated using the following formula: $\text{MPGM score}=\sum \left(\text{mutationstatus}\times \text{coefficient}\right)$. The mutation status was defined as follows: “0” denoted the absence of any mutations, while “1” indicated the presence of mutations. The median cutoff value was set to stratify into MPGM‐high and MPGM‐how groups.

### 2.6 Immune infiltrating analysis of risk model

The relative abundance of immune infiltrating cells was analyzed using a different algorithm, including Cibersort,^20^ Timer,^21^ Quantiseq,^22^ and MCPcounter.^23^ Among them, the result from Cibersort provided a relative abundance of immune cells. EPIC was used to estimate the ratio of immune cells to cancer cells.^24^ The estimate method was utilized to predict tumor purity and stroma abundance and assess the immune cell invasion level.^25^ Additionally, Xcell was employed to evaluate the relative enrichment level of immune cells within the tumor tissue.^26^

### 2.7 Statistical analysis

Fisher's exact test (two‐sided) was used to assess the correlation between clinical pathology features and somatic mutations. Utilizing the chi‐square test, we analyzed differences in the smoking population between two mutation signature groups. Two‐sided Wilcoxon rank sum testing was used between two continuous variables, such as TMB level. Kaplan–Meier survival curves and Cox regression (hazard ratio [HR] and 95% confidence interval [CI]) for survival variables were assessed for the OS and PFS estimations. *p* < 0.05 or FDR < 0.05 were considered statistically significant. The performance of the risk model was assessed by calculating the area under the curve (AUC) of the ROC curve. Statistical analyses were conducted using R software (version 4.1.2).

## 3 RESULTS

### 3.1 Clinical characters of DPH cohort

We retrospectively enrolled 247 Chinese LUAD patients who sought treatment at the Dianjiang People's Hospital (the “DPH cohort,” *N* = 247). We collected comprehensive clinicopathologic features of patients, including age, sex, tumor size, smoking status, body mass index (BMI) index, family history of cancer, ki67 expression, stage, histological classification, histological grade, STAS, lymph node metastasis, vascular invasion, TMB value, and so on, to assess its correlation with genome and survival prognosis. All the tumor tissues of LUAD patients in the DPH cohort underwent an 1123 gene panel target sequencing (ChosenMed, Beijing, China).

### 3.2 Identification of a somatic mutation in LUAD of DPH cohort

**FIGURE 1:**
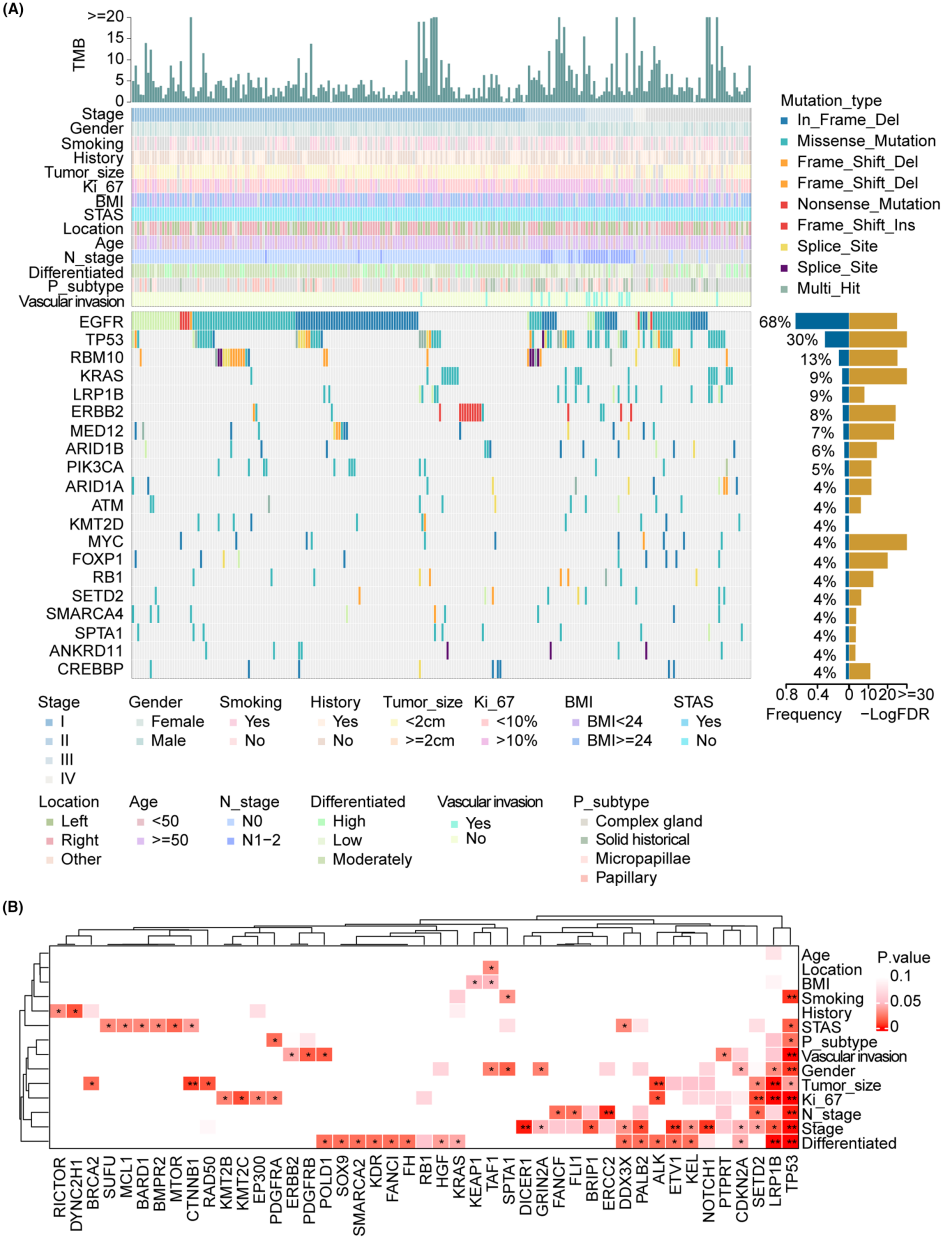
The comprehensive clinical and somatic mutation characteristics of LUAD in the DPH cohort. (A) The mutation landscape of somatic mutations. The upper panel annotations showed the TMB level, the middle panel showed the clinical features, and the lower panel showed the gene mutations, which were colored according to the variant type. The correct label described the percentage of mutation frequency (blue bar) and –LogFDR of significant driver genes (yellow bar). (B) The heatmap showed the correlation between 45 CCRGs and clinical risk factors. CCRGs: Clinical Characteristic‐Related Genes. *p ≤ 0.05; **p ≤ 0.01; ***p ≤ 0.001.

We performed a strict filtering standard to screen truthful mutation sites. A total of 1419 single‐nucleotide variants and 101 insertions/deletions (indels) were called (see “Section 2” and Table S2). The mutation landscape of the top 20 frequently mutant genes and corresponding clinical pathology information is shown in Figure 1A. The lollipop plot demonstrated the mutation position and types of 20 genes (Figure S1). Based on the TMB cutoff value of 10 mutation/Mb, 27 LUAD (10.93%) patients were classified in the TMB‐H group. The median TMB value of the DPH cohort was 3.43 mutation/Mb, and the median TMB of smokers and non‐smokers were 3.95 and 3.29 mutation/Mb, respectively (Table S1).

Driver genes of the DPH LUAD cohort were identified using the MutSigCV method, and the assessed likely driver genes were listed in Table S8. A total of 34 driver genes were identified with q value (*p*‐adjusted) <0.05 (Table S9). The top 5 frequently mutated driver genes are EGFR (68%), TP53 (30.4%), RBM10 (12.6%), KRAS (8.5%), and LRP1B (8.5%) (Table S9). The pathogenic significance of 34 driver genes was searched in the Cancer Gene Census (CGC, https://cancer.sanger.ac.uk/census) database,^27^ 24 of which played a role in cancers. Among these 24 driver genes listed in the CGC, nine have ever been reported in lung cancer. Furthermore, these 24 genes have demonstrated significant involvement as oncogenes (10/34), fusion genes (9/34), and tumor suppressor genes (15/34), highlighting their crucial roles in cancer. The other 10 driver genes were not reported (denoted as NR) before, including SPAT1 (4%), ANKRD11 (3.6%), ERCC3 (2.8%), RAD50 (2.8%), WRN (2.4%), BLM (2.0%), WISP3 (2.0%), CDK8 (1.6%), PAK3 (1.6%), and WEE1 (1.6%), which required additional experiment validation and functional exploration for their clinical significance.

This study defined Clinical Characteristic‐Related Genes (CCRGs) as genes frequently mutated in individuals with higher clinical risk factors. These risk factors include smoking, advanced pathology stage (Stage II−IV), lower historical differential grade, solid historical subtype, age ≥ 50 years old, tumor size ≥2 cm, ki67 expression proportion ≥ 10%, family history of cancers, BMI ≥24, lymph node metastasis, as well as the presence of STAS and vascular invasion. We identified 45 genes significantly associated with higher clinical risk factors (Figure 1B). Frequent mutations in TP53 and SPTA1 were observed in the smoking population, while KEAP1 and TAF1 mutations were associated with a higher BMI index. Patients with STAS pathology exhibited a higher frequency of TP53, MTOR, CTNNB1, BARD1, BMPR2, DDX3X, MCL1, and SUFU mutations. Similarly, patients carrying TP53, ERBB2, PDGFRB, POLD1, and PTPRT mutations were likelier to display the pathology feature of vascular invasion. TP53, LRP1B, SETD2, RAD50, ALK, CTNNB1, and BRCA2 were frequently observed in patients with larger tumors. The expression of ki67 in tumor tissues strongly correlated with mutations in TP53, LRP1B, SETD2, ALK, KMT2C, EP300, KMT2B, and PDGFRA. Lymph node metastasis was closely related to mutations in TP53, SETD2, ERCC2, FANCF, and FLI1, while patients with advanced pathology stage exhibited a higher frequency of TP53, LRP1B, CDKN2A, GRIN2A, DICER1, BRIP1, NOTCH1, AND PALB2 mutations. Furthermore, MTOR has a higher frequency in solid tumor subtypes. These findings suggest a potential association between gene mutations and various clinical pathology features, although further experimental evidence is required to confirm the underlying biological mechanisms. We also retrieved the pathogenic significance of these CCRG genes from the CGC database. Of these, 18 were not reported, and the other 27 played essential roles as oncogenes (12/27), fusion genes (9/27), and tumor suppressor genes (17/27) (Table S9).

We combined the genes of the top 20 frequently mutant genes, 34 driver genes, and 45 CCRGs as essential genes in LUAD patients from the DPH cohort. Seven of these 72 critical genes (ERBB2, KRAS, LRP1B, RB1, SETD2, SPAT1, and TP53) exhibited all three above labels (Table S9). Among the seven genes, SPAT1 was never reported as a driver gene in the CGC database, and LRP1B and SETD2 were reported as driver genes for the other cancer type.

### 3.3 Oncogenic signaling pathway alterations and mutation signature enrichment analysis regulated by somatic mutations in the DPH cohort

**FIGURE 2:**
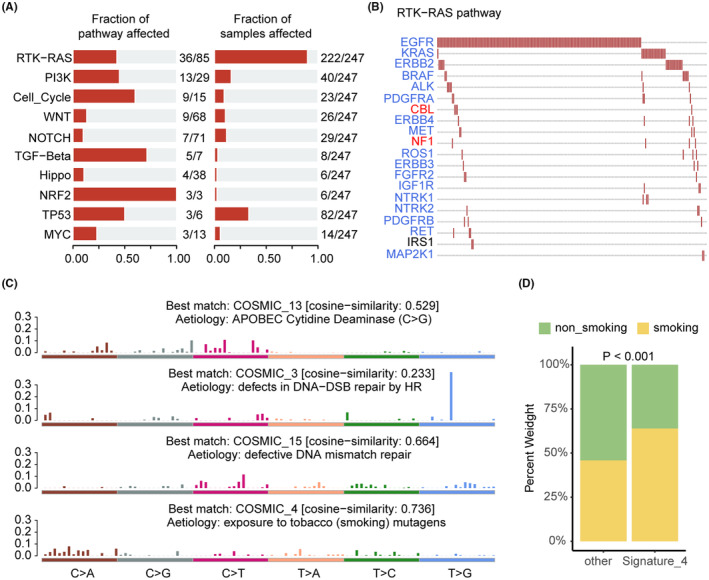
The Oncogenic signaling pathway alterations and mutation signature enrichment analysis regulated by somatic mutations in the DPH cohort. (A) The modifications of 10 oncogenic pathways are based on the mutation data. (B) The gene alterations of the RTK‐RAS pathway. (C) Mutation signature enrichment analysis. (D) The comparison of smoking status between the “smoking” signature group and the rest.

Genetic alterations in signaling pathways have been implicated in heterogeneous tumors' growth and progression processes. In the present study, we analyzed mutations in 10 oncogenic signaling pathways, including RTK‐RAS, NRF2, TP53, PI3K, WNT, Cell Cycle, HIPPO, MYC, NOTCH, TGF‐Beta,^28^ using data from 247 LUAD patients in the DPH cohort. The RTK‐RAS pathway (222/247, 89.88%) and TP53 pathway (82/247, 33.20%) exhibited the highest frequency of pathway alterations (Figure 2A). Among the RTK‐RAS pathway genes, EGFR, KRAS, ERBB2, BRAF, ALK, and PDGFRA were the most frequently altered oncogenes (Figure 2B).

Mutation signature analysis was performed using somatic mutation data of the DPH cohort. We observed four significantly enriched signatures (Figure 2C). The most prevalent signatures were cosmic4 (smoking signature, cosine‐similarity = 0.736) and cosmic15 (defective DNA mismatch repair signature, cosine‐similarity = 0.664). Furthermore, we conducted a comparison of smoking statuses between the “smoking” signature group and the remaining patients. It was observed that the percentage of smokers within the “smoking” signature group was significantly higher compared to the non‐smoking population (*p* < 0.001, Figure 2D).

Overall, alterations in the RTK‐RAS signaling pathway served as the primary oncogenic molecular driver of LUAD patients in the DPH cohort. At the same time, smoking acted as the primary clinical risk characteristic.

### 3.4 Development and validation of Multiple Parameter Gene Mutation (MPGM) risk model

In the present study, we investigated the significant gene alterations observed in the DPH cohort, elucidating the intricate relationship between clinical pathology and the oncogenic implications of gene mutations. Now, we aim to explore the survival prognosis roles of these gene alterations. Due to a lack of adequate follow‐up survival data for the DPH cohort, we used the EAS cohort to develop the risk model. We validated the predictive performance utilizing the MSK‐LUAD cohort.

Seventy‐two critical genes (top 20 frequently mutant genes, 34 driver genes, and 45 CCRGs, Table S9) and 10 oncogenic pathway gene alterations (at least one pathway gene mutated) in the DPH cohort are defined as MPGM for LUAD patients, which were used to screen prognosis‐related signature genes during the initial stage. Genes with mutation frequency = 0% in the EAS cohort were excluded (Figure S2). Finally, 56 essential genes and 10 oncogenic pathway gene alterations were selected for univariate and multivariable Cox regression analysis. Finally, five MPGMs with *p* < 0.05 in univariate and multivariable Cox regression analysis (Table 1). The candidate MPGMs and the coefficients were BRCA2 (1.250), ALK (1.883), BRAF (1.083), EGFR (−0.547), and PDGFRA (0.973). Therefore, the MPGM risk scores were calculated using the following formula: $\text{MPGM score}=\sum \left(\text{mutationstatus}\times \text{coefficient}\right)$. The mutation status was defined as follows: “0” denoted the absence of any mutations, while “1” indicated the presence of mutations. The cutoff was defined as the median value of the MPGM score in the EAS cohort.

**TABLE 1:** Univariable and multivariable analyses of OS of MPGMs of patients in the EAS cohort.

Gene name	Univariate Cox		Multivariate Cox		
	HR (95%CI)	*p*‐value	Coef	HR	*p*‐value
BRCA2	4.708 (2.05–10.80)	0.00026	1.041	2.83 (1.12–7.19)	0.0285
ALK	6.48 (1.58–26.57)	0.0094	1.941	6.966 (1.67–29.16)	0.0079
BRAF	2.944 (1.28–6.75)	0.01	1.056	2.875 (1.23–6.74)	0.0151
EGFR	0.452 (0.29–0.71)	0.00048	−0.536	0.585 (0.37–0.94)	0.0254
PDGFRA	2.717 (1.10–6.72)	0.03	1.142	3.132 (1.23–7.95)	0.0163
KEAP1	5.415 (2.79–10.49)	*p* < 0.0001	1.561	4.762 (0.56–40.67)	0.154
NRF2 pathway	3.113 (1.65–5.86)	*p* = 0.0004	−0.673	0.510 (0.069–3.766)	0.509
PTEN	3.874 (1.69–8.91)	*p* = 0.0014	0.856	2.352 (0.87–6.34)	0.091
PI3K pathway	1.775 (1.18–2.68)	*p* = 0.0061	0.382	1.465 (0.95–2.26)	0.08

Abbreviations: CI: confidence interval; HR, hazard ratio.

**FIGURE 3:**
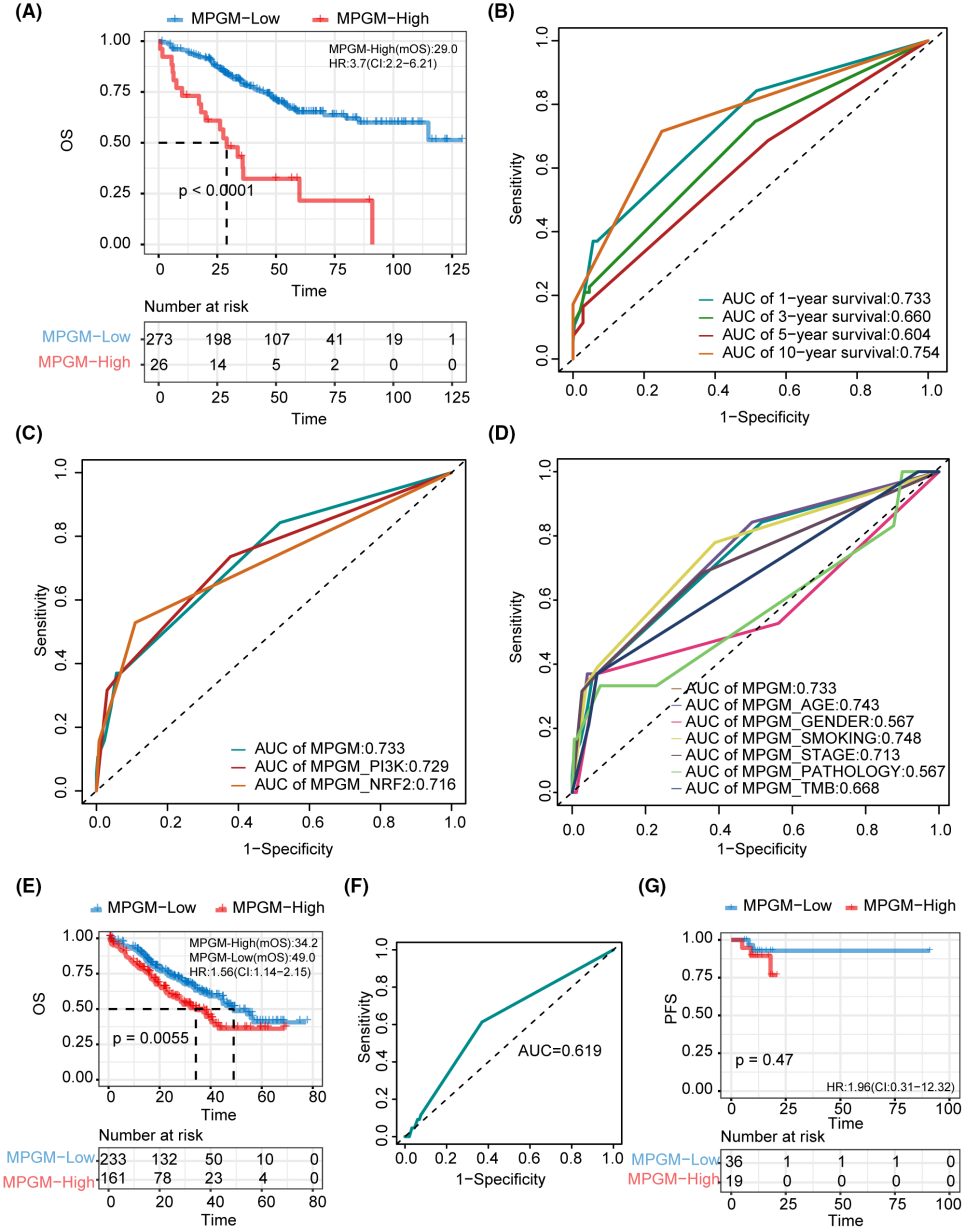
Develop and validate the MPGM risk model. (A) Kaplan–Meier curves depicting OS for patients with MPGM‐high and MPGM‐low scores in the EAS cohort. (B) ROC analysis showed the AUCs of the MPGM risk model in predicting the OS of patients in the EAS cohort. (C) ROC analysis showed the AUCs of the MPGM risk model and the model combined with NRF2 and PI3K pathway alterations in predicting the OS of patients in the EAS cohort. (D) ROC analysis showed the AUCs of the MPG risk model and the model combined with clinical factors in predicting the OS of patients in the EAS cohort. (E) Kaplan–Meier curves of OS for patients with MPGM‐high and MPGM‐low scores in the MSK‐LUAD cohort. (F) ROC analysis showed the AUCs of the MPGM risk model in predicting the OS of patients in the MSK‐LUAD cohort. (G) Kaplan–Meier curves of PFS for 55 patients with MPGM‐high and MPGM‐low scores in the DPH cohort. ROC: receiver operating characteristic; AUC: areas under the ROC curve.

Stratifying 299 patients with LUAD from the EAS cohort into two groups based on the MPGM risk model resulted in MPGM‐high (*N* = 26) and MPGM‐low (*N* = 273) groups (Figure 3A). Survival analysis unveiled a notably favorable prognosis within the MPGM‐low group (*p* < 0.0001, HR = 3.7, 95% CI [2.2, 6.21], median overall survival [mOS]: not reached vs. 29 months). Demonstrating its robustness in outcome prediction, the MPGM risk model achieved the highest AUC of 0.754 for predicting 10‐year survival (Figure 3B). Interestingly, we observed a decreased AUC when integrating the MPGM risk model with two significant pathway alterations in univariate Cox regression analysis: NRF2 and PI3K pathways (Figure 3C). This finding suggested that combining these specific pathway alterations may affect the predictive accuracy of the MPGM risk model.

Moreover, we compare the predicting performance of the MPGM risk model and the model combined with other clinical factors, including age, gender, smoking, stage, histological, and TMB. MPGM risk model combined smoking factor achieved an optimal AUC value of 0.748 (Figure 3D). Given the satisfactory predictive performance achieved by integrating the MPGM risk model and clinical characteristics, we performed the survival analysis by combing the MPGM model and clinical factors. In the MPGM risk model, when age was combined, the MPGM‐high group consistently demonstrated superior survival compared to the MPGM‐low group, regardless of whether the individuals were above or below the age of 64. When the patients in the MPGM‐low group were younger than 64 years old, they achieved the best OS (Figure S3A).

Additionally, this trend persisted irrespective of the TMB value being high or low (Figure S3D) and regardless of smoking status (Figure S3E). These findings highlight the independent predictive capacity of the MPGM risk model. When the MPGM model was combined with the stage (Figure S3B), gender (Figure S3C), and historical differentiation grade (Figure S3F), it could further facilitate a more comprehensive prognostic stratification of patients, thereby aiding clinicians in making personalized clinical decisions.

To validate the predictive efficacy of the MPGM risk model, we utilized the MSK‐LUAD cohort, which provided genomic alterations and survival data. Patients in the MSK‐LUAD cohort were stratified into MPGM‐high and MPGM‐low groups based on the median cutoff value (Figure 3E). Notably, patients in the MPGM‐how group exhibited a slightly prolonged overall survival (OS) benefit (*N* = 233, *p* = 0.0055, HR = 1.56, 95% CI [1.14, 2.51], mOS = 49 months) compared to those in the MPGM‐high group (*N* = 161, mOS = 34 months). The AUC in the validation cohort was 0.619 (Figure 3F). Moreover, we tried to perform the validation in the DPH cohort. By the end of the latest follow‐up visit on 2nd June 2023, the treatment and PFS information of 55 patients from the DPH cohort were collected for validation analysis. As shown in Figure 3G, patients could also be divided into MPGM‐high and MPGM‐low groups despite no significant PFS difference (*p* = 0.47).

Furthermore, we conducted a comparative analysis to elucidate the HLA variability (Figure S4A) and the proportion of distinctive clinical characteristics between the MPGM‐high and MPGM‐how groups (Figure S4B–O). According to the results, Patients in the MPGM‐high group had a significantly high proportion of low differentiation (*p* < 0.01, Figure S4E), expression of ki67 > 10% (*p* = 0.018, Figure S4I), and smokers (*p* = 0.02, Figure S4N).

**FIGURE 4:**
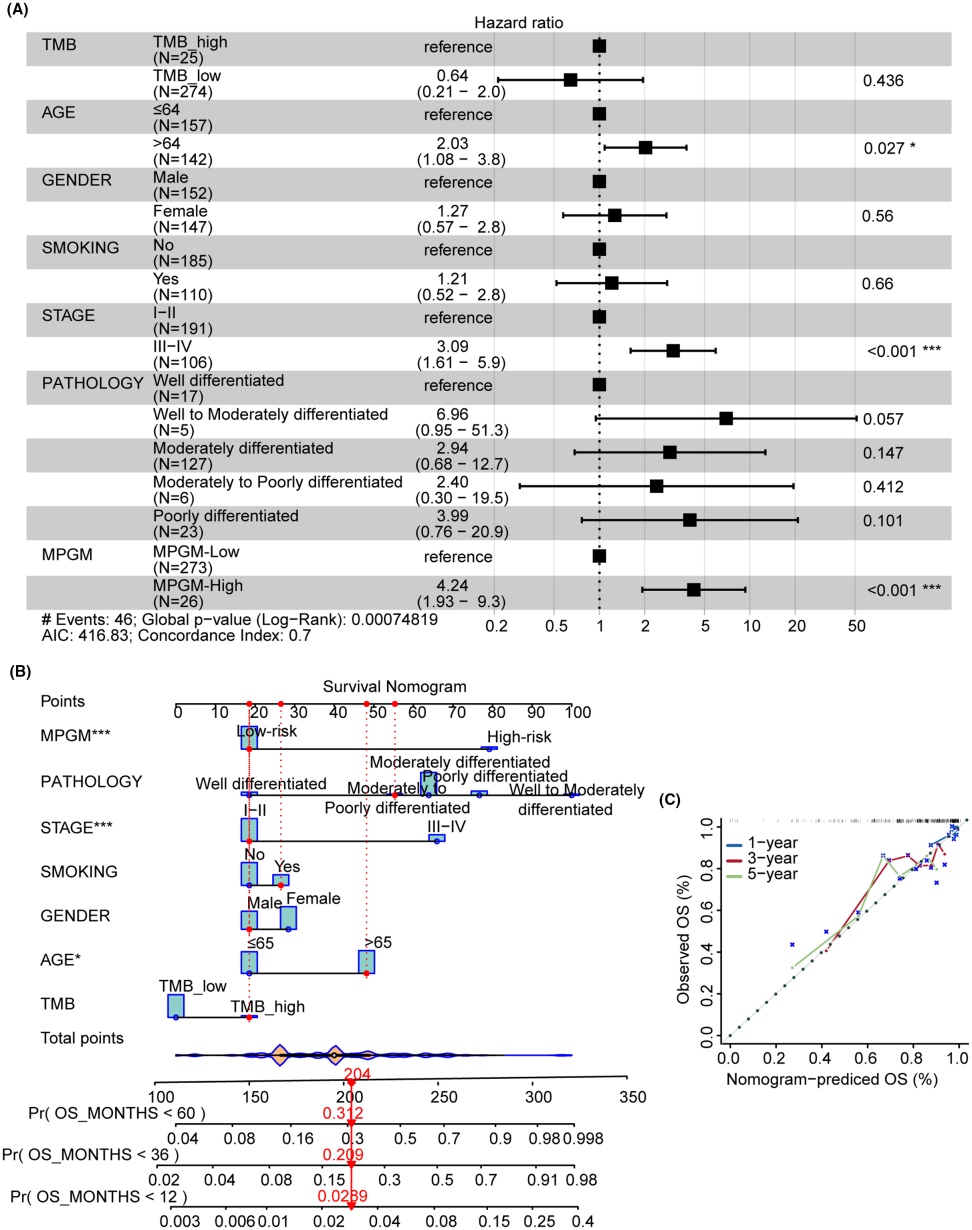
Development of a nomogram model. (A) The forest plot showed the hazard ratio (HR) for OS of the MPGM risk model and cliniophathology factors in the EAS cohort. (B) A nomogram based on histological grade, stage, smoking status, sex, age, TMB, and MPGM risk model for 1‐, 3‐, and 5‐year OS predictions. (C) Calibration curves for testing the agreement between 1‐, 3‐, and 5‐year predicted OS and actual observations in the EAS cohort.

The forest plot demonstrated that age > 64 versus age ≤ 64 (HR = 2.03; 95% CI [1.08–3.8]; *p* = 0.027), stage III‐IV versus stage I–II (HR, 3.00; 95% CI [1.61, 5.9]; *p* < 0.001), and MPGM‐high versus MPGM‐low (*p* < 0.001, HR = 4.24; 95% CI [1.93, 9.3]) were independently correlated with survival prognosis in EAS cohort (Figure 4A). Due to the independence of the MPGM risk model, we constructed a nomogram based on the multivariable Cox regression analysis, and age, histological grade, stage, smoking status, sex, and TMB were incorporated (Figure 4B). The nomogram possessed the integrated effects of the MPGM risk model and clinical factors, which could predict survival for 1‐year, 3‐year, and 5‐year more accurately. The Calibration curves were plotted to show the accuracy of the nomogram in predicting the 2‐, 3‐, and 5‐year survival rates (Figure 4C).

Moreover, a comprehensive comparison was conducted to evaluate the disparities in 10 oncogenic pathway alterations and TMB levels between the MPGM‐high and MPGM‐low groups in EAS and DPH cohorts. The alteration frequency of 10 oncogenic pathways was higher in the MPGM‐low group than in the MPGM‐high group (Figure S5A). However, a significantly higher TMB level was observed in the MPGM‐high group than in the MPGM‐low group, both in the DPH cohort (Figure S5B) and the EAS cohort (Figure S5C). This observation was primarily attributed to the prevalence of sporadic mutations in the MPGM‐low group. In contrast, the MPGM‐high group predominantly exhibits specific carcinogenic gene alterations, which led to higher hazard risk. The detailed gene mutations in the 10 oncogenic signaling pathways of EAS and DPH cohorts are plotted in Figure S6. To assess the therapy decision, we analyzed the targetable gene mutation site referred to in the National Comprehensive Cancer Network guideline.^12^ The results showed ERBB2_20ins, KRAS_G12/13X, and BRAF_V600E mutations were more frequently mutated in the MPGM‐high group, and EGFR mutations, including EGFR_L858R, EGFR_19del, EGFR_20ins, and EGFR_T790M, EGFR_S768I, EGFR_L861Q, and EGFR_G719A have occurred more in the MPGM‐how group (Figure S5D). We conducted an RNA fusion analysis and identified oncogene fusions, including KIF5B‐RET, CCDC6‐RET, EZR‐ROS1, EML4‐ALK, and CD74‐NRG1, in eight patients from the DPH cohort commonly. Notably, all these eight patients belonged to the MPGM‐high group (Table S10). The analysis of copy number variations in the DPH cohort revealed a notable gene amplification (Table S11). Furthermore, we performed a Gene Ontology (GO) enrichment analysis on the five model genes (Figure S7), revealing a significant enrichment of the regulation of the MAPK cascade. This finding highlights the potential molecular mechanism underlying the MPGM risk model.

### 3.5 Prognosis stratification and immune infiltrates analysis for different LUAD subgroups using the MPGM risk model

**FIGURE 5:**
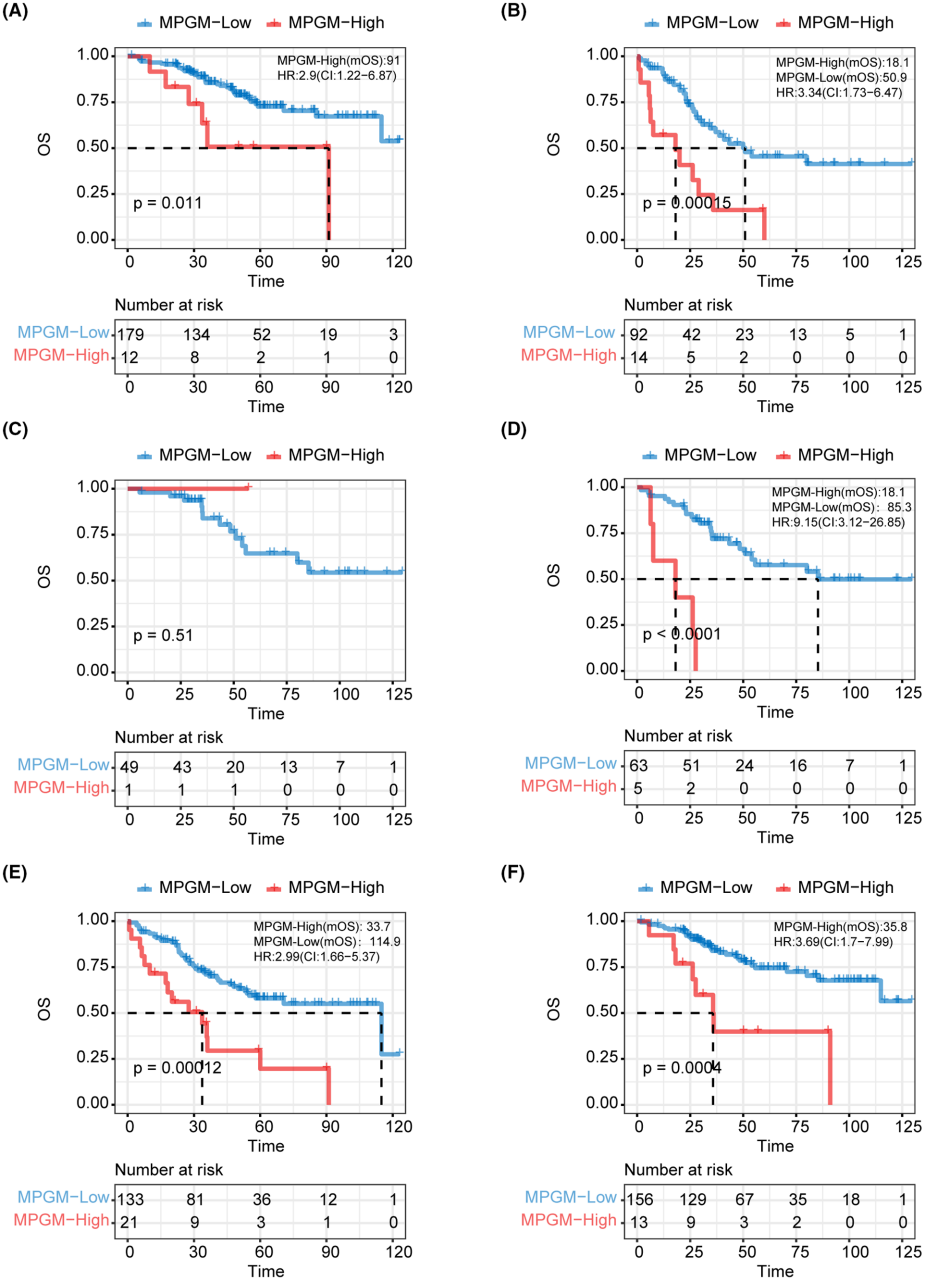
Based on the MPGM risk model, stratified survival analysis was performed according to different clinical application scenarios on the EAS cohort samples. (A) Kaplan–Meier curves of OS for early‐stage LUAD patients (stage I–II, *N* = 191) based MPGM risk model. (B) Kaplan–Meier curves of OS for advanced‐stage patients (stage III–IV, *N* = 106) based MPGM risk model. (C) Kaplan–Meier curves of OS for LUAD patients who received TKIs treatment based MPGM risk model. (D) Kaplan–Meier curves of OS for LUAD patients who undergone chemotherapy (*N* = 68) based MPGM risk model. (E) Kaplan–Meier curves of OS for LUAD patients without EGFR mutations (*N* = 154) based MPGM risk model. (F) Kaplan–Meier curves of OS for 169 LUAD patients with corresponding RNA‐seq data based MPGM risk model.

In addition, we examined the clinical applicability of the MPGM risk model in different LUAD subgroups of the EAS cohort. First, we utilized the MPGM model to stratify early‐stage LUAD patients (stage I–II, *N* = 191), identifying 12 individuals in the MPGM‐high group who require closer follow‐up (*p* = 0.011, HR = 2.9; 95% CI [1.22, 6.87], mOS = 91 months vs. not reached) (Figure 5A). For advanced‐stage patients (stage III–IV, *N* = 106), 92 individuals were classified as MPGM‐low patients using the MPGM risk model (*p* = 0.00015, HR = 3.34; 95% CI [1.73, 6.47], mOS = 50.9 vs. 18.1 months), which needed comprehensive assessments and avoiding overtreatment (Figure 5B). These findings underscore the clinical advantage of the MPGM model in guiding risk‐based decision‐making and optimizing patient management. Subsequently, in the EAS cohort, we identified 50 patients with LUAD who received tyrosine kinase inhibitors (TKIs) treatment. Notably, one patient was classified as an MPGM‐high individual using the MPGM risk model, indicating the potential of the MPGM risk model in identifying treatment hazards (Figure 5C). Following the standard treatment guidelines, patients with EGFR mutations are eligible for corresponding TKI therapy. On the contrary, treatment options are limited for patients without EGFR mutations, and conventional chemotherapy remains the primary choice. We investigated whether the MPGM model could offer additional prognostic stratification for patients without EGFR mutations (*N* = 154). Based on the model, our findings revealed that 21 patients without EGFR mutations were classified into the MPGM‐high group (*p* = 0.00012, HR = 2.99; 95% CI [1.66, 5.37]), which needed risk assessment when considering conventional chemotherapy (Figure 5E).

Furthermore, we investigated whether the MPGM model could be used to identify further patients who are suitable or unsuitable for chemotherapy among those who had undergone chemotherapy (*N* = 68). Five patients with a median OS of 18.1 months were classified into the MPGM‐high group, showing a higher risk when receiving chemotherapy treatment (*p* < 0.0001, Figure 5D). These above findings highlighted the clinical utility of the MPGM risk model in guiding clinical decision‐making regarding drug selection and potentially avoiding adverse outcomes.

Finally, we identified 169 LUAD patients with corresponding RNA‐seq data in the EAS cohort. Survival analysis based on the MPGM model revealed significant differences in OS between the two groups (*p* = 0.0004, Figure 5F). Therefore, we further investigated these two groups' immune microenvironment features based on the RNA‐seq data of 169 LUAD patients in the EAS cohort.

We compared the proportions of immune infiltrating cells in the MPGM‐high and MPGM‐low groups using various immune infiltration analysis methods, including EPIC, Quantiseq, Timer, Xcell, Estimate, Cibersort, and MCPcounter (Figure S8). The ESTIMATE method assessed the proportion of non‐tumor components, including stromal and immune cells. The MPGM‐high group exhibited a higher proportion of tumor purity and a lower ESTIMATEScore, indicating a higher degree of tumor invasiveness. Conversely, the MPGM‐low group showed significant enrichment of dendritic cells (DC) and monocytic lineage cells and a higher MicroenvironmentScore and ImmuneScore (Figure S8), suggesting an enhanced immune anti‐tumor activity.

## 4 DISCUSSION

The latest advance of phase 3 trials ADAURA study (NCT02511106) confirmed Adjuvant osimertinib could get a significant 85% 5‐year OS benefit among patients with completely resected, EGFR‐mutated, stage IB to IIIA NSCLC, showing the powerful therapy efficiency of targeted therapy.^29^ Recent clinical trial research on perioperative immunotherapy in early‐stage NSCLC, including phase 3 trials such as CheckMate 816 and KEYNOTE‐671, has yielded remarkable advancements. These findings signify a paradigm shift in research focus from exploring survival benefits solely in advanced metastatic NSCLC to encompassing the early and mid‐stage NSCLC patients and establishing a new perioperative treatment pattern.^30^, ^31^ Despite significant advancements in clinical trials for targeted therapy and immunotherapy, leading to improved outcomes in LUAD patients, a subset of patients cannot undergo surgery or lack targetable biomarkers. Therefore, it is imperative to explore effective biomarkers to stratify patients and identify additional targetable biomarkers.^32^ Consequently, our objective is to identify tumor‐specific informative genes based on mutation characteristics, facilitating risk stratification for patients, particularly with limited treatment options.

In this study, we retrospectively collected a moderate‐size LUAD cohort and profiled the somatic altered features based on the genomic data of multiple gene sequencing panels. The alteration of the RTK‐RAS pathway and smoking are identified as the main carcinogenic factors. We first explore the association between somatic tumor mutations and clinical risk factors. A total of 45 CCRGs are significantly closely related to the high‐risk clinicopathological characteristics, including smoking, advanced pathology stage (stage II−IV), lower historical differential grade, solid historical subtype, age ≥ 50 years old, tumor size ≥2 cm, ki67 expression proportion ≥ 10%, family history of cancers, BMI index ≥24, lymph node metastasis, as well as the presence of STAS and vascular invasion. The TP53 mutation is significantly associated with tobacco consumption.^33^ In addition, TP53 mutations are substantially higher in patients with other high‐risk clinical factors, including STAS+, high ki67 expression, larger tumor size, and lymph node metastasis. The TP53 mutation in tumors could result in the loss of tumor‐suppressing function, promoting tumor proliferation and metastasis.^34^ Regarding STAS, Ye et al. conducted a study to investigate the molecular characteristics distinguishing NSCLC patients with STAS+ and STAS−. Their findings revealed significant TP53 mutation and ALK fusion occurrences in STAS+ NSCLC patients.^35^ In our study, we also observed a correlation between STAS+ and several oncogene mutations, namely MTOR and CTNNB1, as well as tumor suppressor genes, including BARD1, DDX3X, and SUFU. However, recurrent mutations of EGFR and KRAS were not found to be associated with STAS+, which is consistent with previous studies.^36^, ^37^ KEAP1 is a tumor suppressor gene in NSCLC, which is associated with a higher BMI index of LUAD patients in our study. There is no reported relationship between KEAP1 and BMI index. Still, we found KEAP1 mutation and BMI index are proven to be related to survival benefits from immunotherapy in NSCLC, which provides new insight for further study.^38^, ^39^ Significant associations were observed between vascular invasion and mutations in oncogenes ERBB2 and PDGFRA, as well as tumor suppressor genes POLD1 and PTPRT. PDGFRA is an angiogenesis‐related gene, and the mRNA expression of PDGFRA and vascular invasion are positively related to HIF‐1alpha in hepatocellular carcinoma (HCC), which could promote tumor inflammation.^40^ Furthermore, mutations in tumor suppressor genes LRP1B, SETD2, and TP53 are strongly associated with larger tumor size, elevated ki67 expression, lymph node metastasis, and advanced pathological stage in patients with LUAD, implying a significant association with proliferation and metastasis of LUAD.

Kadara et al. discovered a correlation between SETD2 mutations and the progression as well as poor survival of patients with LUAD.^41^ Additionally, LRP1B mutations have been found to significantly impact the Cell Cycle and Antigen Processing and Presentation pathways, which play crucial roles in tumorigenesis. Notably, NSCLC patients with LRP1B mutations exhibit high TMB values and derive incredible survival benefits from immunotherapy.^42^ These CCRG genes provide new insight into the molecular mechanisms underlying the occurrence of high‐risk clinical factors, thus providing valuable avenues for further investigation in this area of research.

In addition to identifying gene mutations significantly associated with these clinical features, we also identified novel driver genes, including SPAT1, ANKRD11, ERCC3, RAD50, WRN, BLM, WISP3, CDK8, PAK3, and WEE1, which have not been reported in the Cancer Gene Census (CGC) database. Further experimental validation is required to elucidate their biological mechanisms in LUAD.

To assess the prognostic significance of LUAD‐related genomic alterations, including CCRGs, driver genes, and gene mutations in oncogene pathways, we developed an MPGM risk model using a multivariable Cox regression algorithm in a publicly available Chinese LUAD cohort (EAS cohort). Patients stratified into two groups, MPGM‐high and MPGM‐low, by the risk model, showed inferior OS compared to those in the MPGM‐low group, as validated in an independent cohort (MSK‐LUAD cohort). Subgroup analysis shows the robust predicting performance of the MPGM model to stratify the LUAD patients. The MPGM risk model could effectively distinguish LUAD patients of the EAS cohort into MPGM‐high and MPGM‐low groups, irrespective of EGFR mutation status, treatment type (chemotherapy or targeted therapy), and disease stage (early or advanced). Remarkable survival differences were observed between the two risk groups, demonstrating the model's clinically solid applicability and stable prognostic performance.

Although statistical significance was not achieved in the DPH cohort, an analysis of 54 patients with complete PFS follow‐up information revealed that those in the MPGM‐low group exhibited better PFS than those in the MPGM‐high group. Additionally, several characteristics were observed in the MPGM‐high group, including higher ki67 expression, smoking history, lower differentiation grade, high TMB, and specific gene mutations in the RTK‐RAS pathway. Significant differences were also observed in targetable gene mutation sites for targeted therapy. The MPGM‐high group showed a higher prevalence mutation of ERBB2_20ins, KRAS_G12/13X, and BRAF_V600E mutations, while the MPGM‐low group had a higher occurrence of EGFR_L858R, EGFR_19del, EGFR_20ins, and EGFR_T790M, EGFR_S768I, EGFR_L861Q, and EGFR_G719A mutations. Interestingly, it was observed that all patients harboring detected oncogenic fusion genes, including RET, ROS1, ALK, and NRG1,^12^ were consistently classified into the MPGM‐high group. These findings provide compelling evidence that patients classified as MPGH‐high risk exhibit a higher risk hazard, both in clinical and molecular characteristics. Moreover, these results emphasize the model's substantial clinical utility and robust prognostic capacity in real‐world settings. Due to the favorable clinical application of the MPGM risk model and improved prognosis assessment performance when combined with the clinicopathology feature, we establish a nomogram based on the Cox regression model, which is pretty valuable for the clinics to precisely predict the survival status using the clinical information at baseline and MPGM risk model according to the molecular biological characteristics, including three CCRGs (BRCA2, ALK, and PDGFRA) and two driver genes (BRAF, EGFR). These five genes all play essential roles in LUAD.

Analyzing immune infiltration differences using RNA‐seq data, we sought to understand the reasons behind the disparate survival outcomes between the MPGM‐high and MPGM‐low groups. It was observed that patients in the MPGM‐high group exhibited higher tumor purity and lower ESTIMATE score, indicative of increased tumor cell infiltration and decreased immune cell enrichment.^25^ Patients in the MPGM‐low group showed significant enrichment of dendritic cells (DC) and monocytic lineage cells and a higher MicroenvironmentScore and ImmuneScore, demonstrating robust immune activation and anti‐tumor capabilities. A previous study has confirmed that tumor‐associated DC is related to increased survival outcomes in lung cancer due to an increased anti‐tumor T‐cell response.^43^, ^44^ The monocytic lineage cells could differentiate into macrophage cells, affecting tumor growth and survival outcomes.^45^ The monocyte lineage and macrophage cells demonstrate a significant interaction in LUAD, correlating with unfavorable survival outcomes and high‐grade tumor subtypes.^46^ But, this disadvantage may be negated by heightened immune activity in the MPGM‐low group.

In summary, the significantly higher anti‐tumor capability and better survival of the MPGM‐low group can mainly be attributed to the infiltration of immune cells and DCs. In contrast, the MPGM‐high group exhibits extensive invasion and infiltration of tumor cells, resulting in a markedly suppressive tumor microenvironment and compromised survival prognosis. These findings reinforce the reliable and robust predictive performance of our model, highlighting its credibility and resilience.

We will apply the MPGH risk model to our clinical practice. For these 55 LUAD patients who keep a close follow‐up in the DPH cohort, patients in the MPGM‐high group will be paid extra attention to the disease progression and gene alterations during the subsequent follow‐up. In contrast, patients in the MPGM‐low group will undergo the standard therapy method and avoid overtreatment.

Our study has several limitations. First, the DPH cohort predominantly comprises early‐stage LUAD patients, resulting in a loss of follow‐up for many individuals. As a result, only 55 patients have adhered to regular follow‐up visits. Second, in the DPH cohort, the MPGM risk model did not demonstrate a statistically significant difference between the MPGM‐high and MPGM‐low groups. This lack of significance can be attributed to the relatively short follow‐up period and the fact that only a minority of patients experienced disease progression. Due to the limited number of samples available for follow‐up, we were unable to conduct further analysis on the impact of gene mutation status and treatment modalities on prognosis. Nevertheless, we remain committed to persistently conducting follow‐ups for this specific group of individuals.

In addition, genetic intratumor heterogeneity is closely linked to tumor occurrence, development, and overall outcome. Mutant‐allele fraction (MAF) serves as a valuable metric for inferring tumor heterogeneity and purity, and its level has been implicated in influencing cancer prognosis, particularly in NSCLC.^47^

Given the potential impact of MAF on tumor behavior and patient prognosis, its role in affecting the performance of predictive models like ours warrants further exploration. As we move forward with our research endeavors, we recognize the importance of integrating MAF into our analytical framework to comprehensively understand its mechanisms of influence and its implications for patient care and management.

## 5 CONCLUSIONS

In addition to exploring therapeutic targets for targeted therapy opportunities, it is essential to consider patient stratification based on prognosis. Beyond utilizing clinical and pathological features like TNM staging and histological subtyping, leveraging patient‐specific molecular alterations is crucial for precise risk stratification of heterogeneous patients, enabling personalized diagnostic and therapeutic assessments. In this study, we comprehensively investigated the molecular characteristics of LUAD and identified several novel putative driver genes and genes associated with clinical pathological characteristics. These findings provide new insight for studying molecular biological features and drug development for patients with LUAD. By assessing the risk level of LUAD patients using the MPGM risk model, we can make informed decisions regarding treatment selection. For patients classified into the MPGM‐low group, a thorough evaluation will determine if they can be spared from excessive treatments, reducing unnecessary suffering. Conversely, the MPGM‐high group LUAD patients in advanced stages may benefit from more comprehensive evaluations, allowing for the exploration of more effective treatment options, such as immunotherapy. By utilizing patient‐specific genetic alteration data, we can not only identify actionable targets for targeted therapy but also predict their survival prognosis. Ultimately, this risk model has the potential to improve patient outcomes by providing personalized treatment strategies.

## AUTHOR CONTRIBUTIONS

**Tongxin Li:** Conceptualization (equal); formal analysis (lead); writing – original draft (equal); writing – review and editing (equal). **Jie Liu:** Conceptualization (equal); formal analysis (equal); writing – original draft (equal). **Yu Zhou:** Formal analysis (equal). **Shengyuan Huang:** Data curation (lead); formal analysis (supporting); visualization (equal). **Dong Wang:** Data curation (equal); visualization (equal). **Jianrong Chen:** Data curation (equal). **Yong Fu:** Conceptualization (equal); supervision (equal); writing – review and editing (equal). **Ping He:** Conceptualization (equal); funding acquisition (equal); supervision (equal); writing – review and editing (equal).

## FUNDING INFORMATION

This work was supported by grants from Dianjiang County Science and Technology Bureau of Chongqing (no. djkjxm2021shmskjcxyw002), Chongqing Medical Scientific Research Project (Joint project of Chongqing Health Commission and Science and Technology Bureau) (2024MSXM054), and the Natural Science Foundation of Chongqing (cstc2020jcyj‐msxmX1060).

## CONFLICT OF INTEREST STATEMENT

All authors declare that the research was conducted without any commercial or financial relationships that could be construed as a potential conflict of interest.

## ETHICAL APPROVAL

This study was approved by the ethics committee of Dianjiang People's Hospital of Chongqing (Number 20220906).

## CONSENT TO PARTICIPATE

Informed consent was obtained from all individual participants included in the study.

## CONSENT TO PUBLISH

Not applicable.

## Supporting information


Figures S1–S8.

Tables S1–S11.

## Data Availability

The analysis results data generated during this study are in supplementary tables.

## References

[1] Sung H , Ferlay J , Siegel RL , et al. Global cancer statistics 2020: GLOBOCAN estimates of incidence and mortality worldwide for 36 cancers in 185 countries. CA Cancer J Clin. 2021;71(3):209‐249. 33538338 10.3322/caac.21660

[2] Herbst RS , Morgensztern D , Boshoff C . The biology and management of non‐small cell lung cancer. Nature. 2018;553(7689):446‐454. 29364287 10.1038/nature25183

[3] Mitsudomi T , Tan D , Yang JC‐H , et al. Expert consensus recommendations on biomarker testing in metastatic and nonmetastatic NSCLC in Asia. J Thorac Oncol. 2023;18(4):436‐446. 36379356 10.1016/j.jtho.2022.10.021

[4] Tu H , Ye Y , Huang M , et al. Smoking, smoking cessation, and survival after cancer diagnosis in 128,423 patients across cancer types. Cancer Commun (London). 2022;42(12):1421‐1424. 10.1002/cac2.12357 PMC9759761 36042006

[5] Chen P , Liu Y , Wen Y , Zhou C . Non‐small cell lung cancer in China. Cancer Commun (London). 2022;42(10):937‐970. 10.1002/cac2.12359 PMC9558689 36075878

[6] Guerreiro T , Forjaz G , Antunes L , et al. Lung cancer survival and sex‐specific patterns in Portugal: a population‐based analysis. Pulmonology. 2023;29(Suppl 4):S70‐S90. 34642125 10.1016/j.pulmoe.2021.09.001

[7] Lee GY , Chung J‐H , Cho S , et al. Impact of preoperative diagnostic biopsy procedure on spread through airspaces and related outcomes in resected stage I non‐small cell lung cancer. Chest. 2022;162(5):1199‐1212. 35562060 10.1016/j.chest.2022.05.002

[8] Han YB , Kim H , Mino‐Kenudson M , et al. Tumor spread through air spaces (STAS): prognostic significance of grading in non‐small cell lung cancer. Mod Pathol. 2021;34(3):549‐561. 33199839 10.1038/s41379-020-00709-2

[9] Martínez‐Jiménez F , Muiños F , Sentís I , et al. A compendium of mutational cancer driver genes. Nat Rev Cancer. 2020;20(10):555‐572. 32778778 10.1038/s41568-020-0290-x

[10] Tamborero D , Rubio‐Perez C , Deu‐Pons J , et al. Cancer genome interpreter annotates the biological and clinical relevance of tumor alterations. Genome Med. 2018;10(1):25. 29592813 10.1186/s13073-018-0531-8 PMC5875005

[11] Network CGAR . Comprehensive molecular profiling of lung adenocarcinoma. Nature. 2014;511(7511):543‐550. 25079552 10.1038/nature13385 PMC4231481

[12] Ettinger DS , Wood DE , Aisner DL , et al. NCCN guidelines® insights: non–small cell lung cancer, Version 2.2023: featured updates to the NCCN guidelines. J Natl Compr Cancer Netw. 2023;21(4):340‐350. 10.6004/jnccn.2023.0020 37015337

[13] Detterbeck FC , Boffa DJ , Kim AW , Tanoue LT . The eighth edition lung cancer stage classification. Chest. 2017;151(1):193‐203. 27780786 10.1016/j.chest.2016.10.010

[14] Li Q , Wang R , Yang Z , et al. Molecular profiling of human non‐small cell lung cancer by single‐cell RNA‐seq. Genome Med. 2022;14(1):87. 35962452 10.1186/s13073-022-01089-9 PMC9375433

[15] Chen J , Yang H , Teo ASM , et al. Genomic landscape of lung adenocarcinoma in east Asians. Nat Genet. 2020;52(2):177‐186. 32015526 10.1038/s41588-019-0569-6

[16] Nguyen B , Fong C , Luthra A , et al. Genomic characterization of metastatic patterns from prospective clinical sequencing of 25,000 patients. Cell. 2022;185(3):563‐575.e11. 35120664 10.1016/j.cell.2022.01.003 PMC9147702

[17] McKenna A , Hanna M , Banks E , et al. The genome analysis toolkit: a MapReduce framework for analyzing next‐generation DNA sequencing data. Genome Res. 2010;20(9):1297‐1303. 20644199 10.1101/gr.107524.110 PMC2928508

[18] Koboldt DC , Zhang Q , Larson DE , et al. VarScan 2: somatic mutation and copy number alteration discovery in cancer by exome sequencing. Genome Res. 2012;22(3):568‐576. 22300766 10.1101/gr.129684.111 PMC3290792

[19] Lawrence MS , Stojanov P , Polak P , et al. Mutational heterogeneity in cancer and the search for new cancer‐associated genes. Nature. 2013;499(7457):214‐218. 23770567 10.1038/nature12213 PMC3919509

[20] Chen B , Khodadoust MS , Liu CL , Newman AM , Alizadeh AA . Profiling tumor infiltrating immune cells with CIBERSORT. Methods Mol Biol. 2018;1711:243‐259. 29344893 10.1007/978-1-4939-7493-1_12 PMC5895181

[21] Li T , Fan J , Wang B , et al. TIMER: a web server for comprehensive analysis of tumor‐infiltrating immune cells. Cancer Res. 2017;77(21):e108‐e110. 29092952 10.1158/0008-5472.CAN-17-0307 PMC6042652

[22] Finotello F , Mayer C , Plattner C , et al. quanTIseq: quantifying immune contexture of human tumors. *BioRxiv* . 2017 223180.

[23] Becht E , Giraldo NA , Lacroix L , et al. Estimating the population abundance of tissue‐infiltrating immune and stromal cell populations using gene expression. Genome Biol. 2016;17(1):218. 27765066 10.1186/s13059-016-1070-5 PMC5073889

[24] Yeo JG , Wasser M , Kumar P , et al. The extended Polydimensional immunome characterization (EPIC) web‐based reference and discovery tool for cytometry data. Nat Biotechnol. 2020;38(6):679‐684. 32440006 10.1038/s41587-020-0532-1

[25] Yoshihara K , Shahmoradgoli M , Martínez E , et al. Inferring tumour purity and stromal and immune cell admixture from expression data. Nat Commun. 2013;4:2612. 24113773 10.1038/ncomms3612 PMC3826632

[26] Aran D , Hu Z , Butte AJ . xCell: digitally portraying the tissue cellular heterogeneity landscape. Genome Biol. 2017;18(1):220. 29141660 10.1186/s13059-017-1349-1 PMC5688663

[27] Sondka Z , Bamford S , Cole CG , Ward SA , Dunham I , Forbes SA . The COSMIC cancer gene census: describing genetic dysfunction across all human cancers. Nat Rev Cancer. 2018;18(11):696‐705. 30293088 10.1038/s41568-018-0060-1 PMC6450507

[28] Sanchez‐Vega F , Mina M , Armenia J , et al. Oncogenic signaling pathways in the cancer genome atlas. Cell. 2018;173(2):321‐337.e10. 29625050 10.1016/j.cell.2018.03.035 PMC6070353

[29] Tsuboi M , Herbst RS , John T , et al. Overall survival with osimertinib in resected EGFR‐mutated NSCLC. N Engl J Med. 2023;389:137‐147. 37272535 10.1056/NEJMoa2304594

[30] Wakelee H , Liberman M , Kato T , et al. Perioperative pembrolizumab for early‐stage non‐small‐cell lung cancer. N Engl J Med. 2023;389:491‐503. 37272513 10.1056/NEJMoa2302983 PMC11074923

[31] Forde PM , Spicer J , Lu S , et al. Neoadjuvant nivolumab plus chemotherapy in Resectable lung cancer. N Engl J Med. 2022;386(21):1973‐1985. 35403841 10.1056/NEJMoa2202170 PMC9844511

[32] Sun L , Bleiberg B , Hwang W‐T , et al. Association between duration of immunotherapy and overall survival in advanced non‐small cell lung cancer. JAMA. Oncologia. 2023;9:1075. 10.1001/jamaoncol.2023.1891 PMC10240399 37270700

[33] Le Calvez F , Mukeria A , Hunt JD , et al. TP53 and KRAS mutation load and types in lung cancers in relation to tobacco smoke: distinct patterns in never, former, and current smokers. Cancer Res. 2005;65(12):5076‐5083. 15958551 10.1158/0008-5472.CAN-05-0551

[34] Alvarado‐Ortiz E , de la Cruz‐López KG , Becerril‐Rico J , Sarabia‐Sánchez MA , Ortiz‐Sánchez E , García‐Carrancá A . Mutant p53 gain‐of‐function: role in cancer development, progression, and therapeutic approaches. Front Cell Dev Biol. 2020;8:607670. 33644030 10.3389/fcell.2020.607670 PMC7905058

[35] Ye R , Yu Y , Zhao R , Han Y , Lu S . Comprehensive molecular characterizations of stage I–III lung adenocarcinoma with tumor spread through air spaces. Front Genet. 2023;14:1101443. 36816028 10.3389/fgene.2023.1101443 PMC9932204

[36] Zeng Q , Wang B , Li J , et al. Solid nodule appearance as a predictor of tumor spread through air spaces in patients with lung adenocarcinoma: a propensity score matching study. Cancer Manag Res. 2020;12:8197‐8207. 32982416 10.2147/CMAR.S266750 PMC7490081

[37] Zhang Z , Liu Z , Feng H , et al. Predictive value of radiological features on spread through air space in stage cIA lung adenocarcinoma. J Thorac Dis. 2020;12(11):6494‐6504. 33282351 10.21037/jtd-20-1820 PMC7711360

[38] Kichenadasse G , Miners JO , Mangoni AA , Rowland A , Hopkins AM , Sorich MJ . Association between body mass index and overall survival with immune checkpoint inhibitor therapy for advanced non‐small cell lung cancer. JAMA Oncol. 2020;6(4):512‐518. 31876896 10.1001/jamaoncol.2019.5241 PMC6990855

[39] Di Federico A , De Giglio A , Parisi C , Gelsomino F . STK11/LKB1 and KEAP1 mutations in non‐small cell lung cancer: prognostic rather than predictive? Eur J Cancer. 2021;157:108‐113. 34500370 10.1016/j.ejca.2021.08.011

[40] Dai C‐X , Gao Q , Qiu S‐J , et al. Hypoxia‐inducible factor‐1 alpha, in association with inflammation, angiogenesis and MYC, is a critical prognostic factor in patients with HCC after surgery. BMC Cancer. 2009;9:418. 19948069 10.1186/1471-2407-9-418 PMC2797816

[41] Kadara H , Choi M , Zhang J , et al. Whole‐exome sequencing and immune profiling of early‐stage lung adenocarcinoma with fully annotated clinical follow‐up. Ann Oncol. 2017;28(1):75‐82. 27687306 10.1093/annonc/mdw436 PMC5982809

[42] Chen H , Chong W , Wu Q , Yao Y , Mao M , Wang X . Association of LRP1B mutation with tumor mutation burden and outcomes in melanoma and non‐small cell lung cancer patients treated with immune check‐point blockades. Front Immunol. 2019;10:1113. 31164891 10.3389/fimmu.2019.01113 PMC6536574

[43] Broz ML , Binnewies M , Boldajipour B , et al. Dissecting the tumor myeloid compartment reveals rare activating antigen‐presenting cells critical for T cell immunity. Cancer Cell. 2014;26(5):638‐652. 25446897 10.1016/j.ccell.2014.09.007 PMC4254577

[44] Guilliams M , Dutertre C‐A , Scott CL , et al. Unsupervised high‐dimensional analysis aligns dendritic cells across tissues and species. Immunity. 2016;45(3):669‐684. 27637149 10.1016/j.immuni.2016.08.015 PMC5040826

[45] Ugel S , Canè S , De Sanctis F , Bronte V . Monocytes in the tumor microenvironment. Annu Rev Pathol. 2021;16:93‐122. 33497262 10.1146/annurev-pathmechdis-012418-013058

[46] Sorin M , Rezanejad M , Karimi E , et al. Single‐cell spatial landscapes of the lung tumour immune microenvironment. Nature. 2023;614(7948):548‐554. 36725934 10.1038/s41586-022-05672-3 PMC9931585

[47] Shen S , Wei Y , Zhang R , et al. Mutant‐allele fraction heterogeneity is associated with non‐small cell lung cancer patient survival. Oncol Lett. 2018;15(1):795‐802. 29399148 10.3892/ol.2017.7428 PMC5772758

