# Supplementary material for: Clinical relevance of somatic mutations in Chinese lung adenocarcinoma and their prognostic implications for survival
Source: Cancer Med. 2024 May 21;13(10):e7227. doi: 10.1002/cam4.7227 (PMC11106684; doi:10.1002/cam4.7227)
Supplement: Supplementary file 1 — Figures S1–S8. [file CAM4-13-e7227-s002.docx]

Supplementary Figures


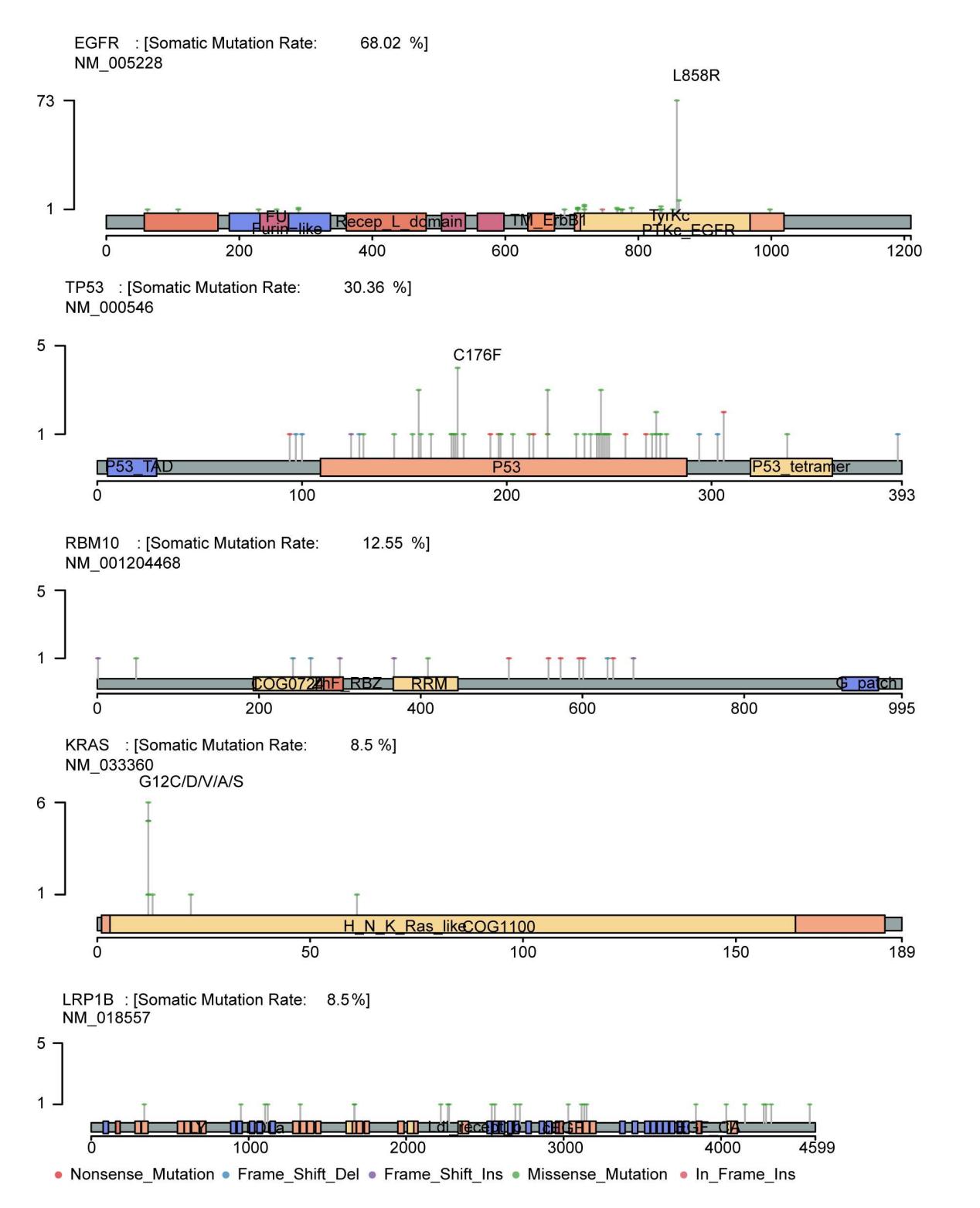

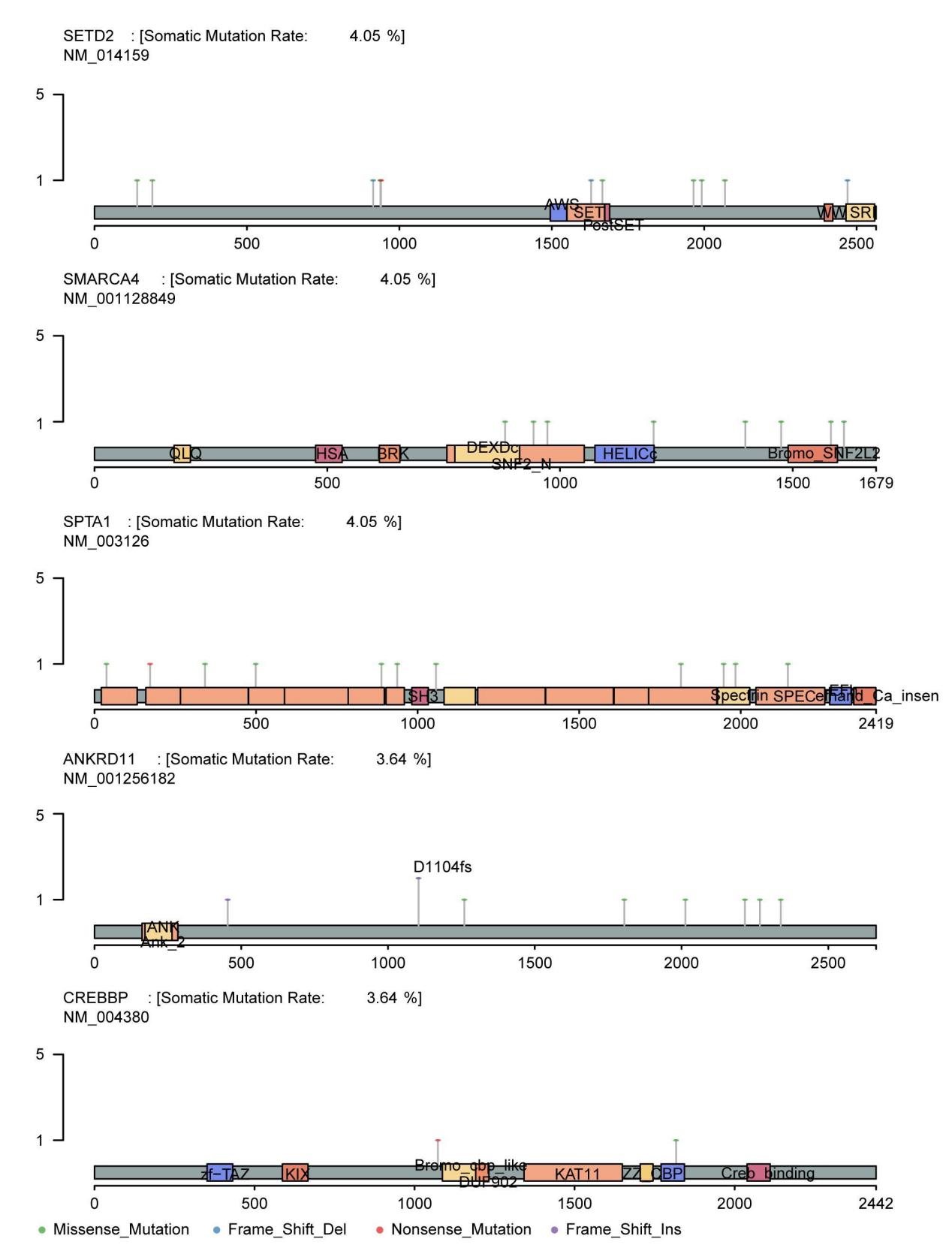


**Figure S1. Lollipop plot showed somatic mutations of the DPH cohort's 20 most frequently altered genes.** lollipop plot indicated each gene's amino acid changes in the protein region (grey bar). Protein motifs were shown with colored boxes.


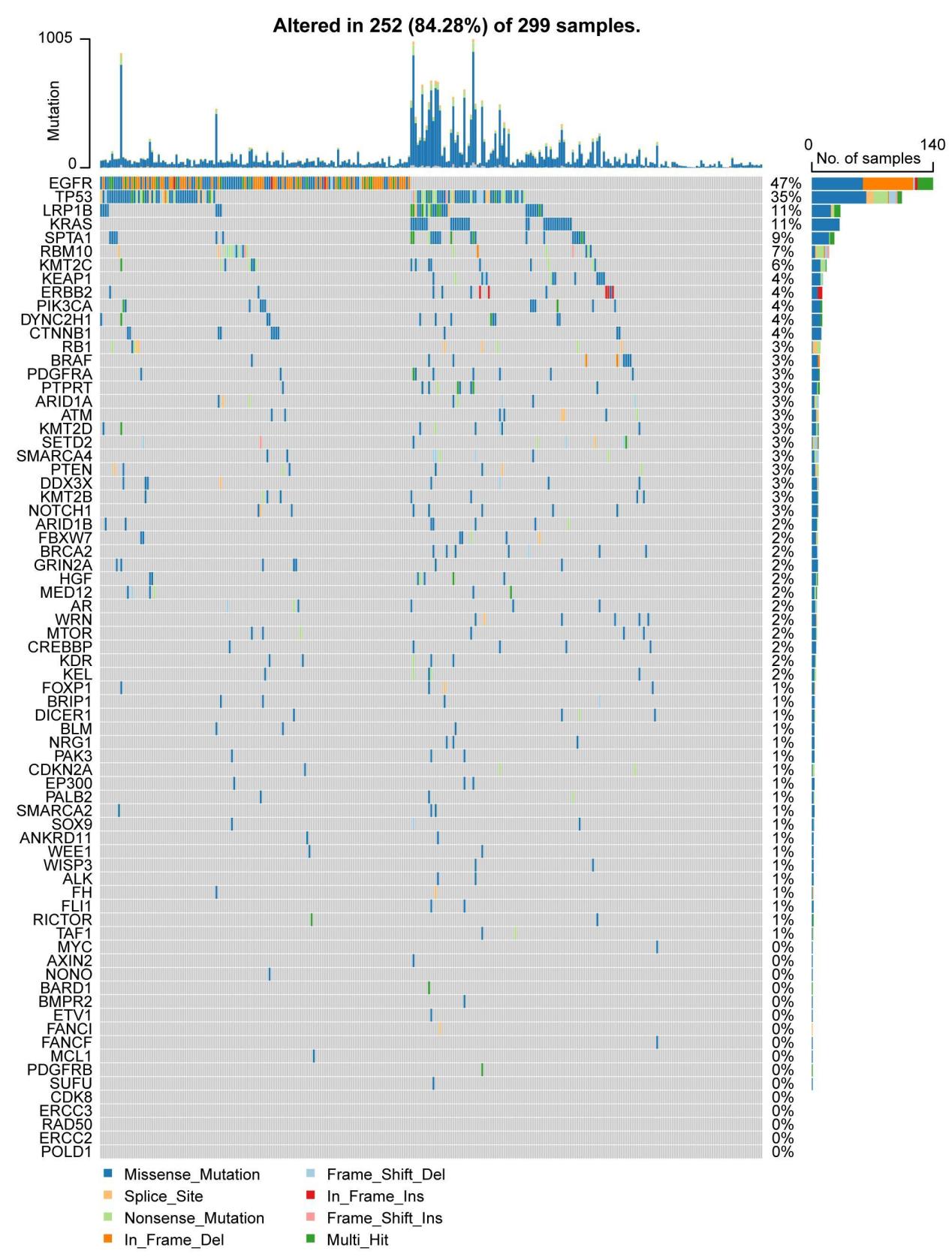


**Figure S2.** **The mutation landscape of 72 critical genes (top 20 frequently mutant genes, 34 driver genes, and 45 CCRGs) in the EAS cohort.**


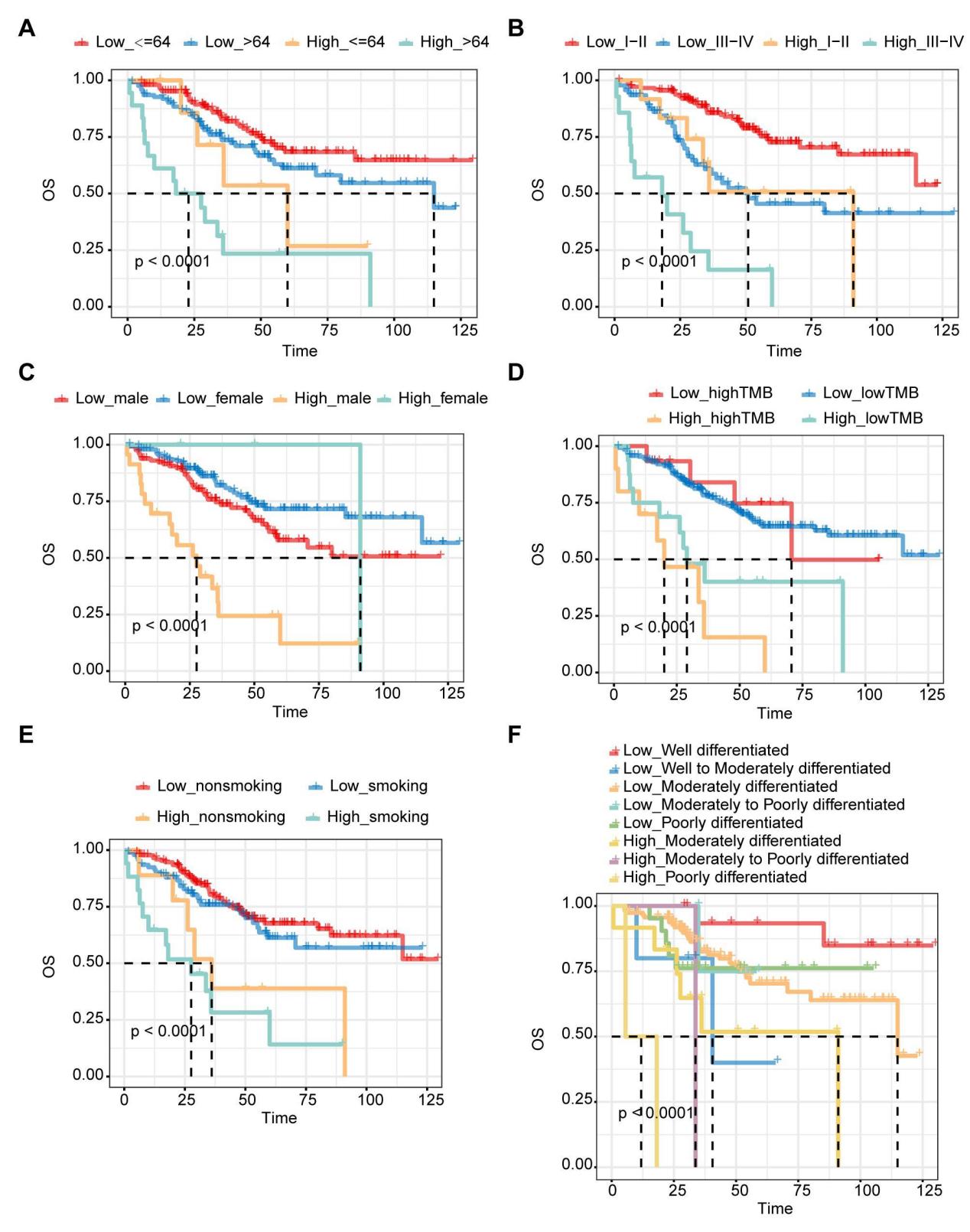


**Figure S3.** **Survival analysis was conducted by integrating the MPGM model with multiple clinical risk factors in the EAS cohort.** (A) Kaplan–Meier curves of OS for patients with different MPGM risk and age groups. (B) Kaplan–Meier curves of OS for patients with different MPGM risk and stage groups. (C) Kaplan–Meier curves of OS for patients with different MPGM risk and gender groups. (D) Kaplan–Meier curves of OS for patients with different MPGM risk and TMB groups. (E) Kaplan–Meier curves of OS for patients with different MPGM risk and smoking groups. (F) Kaplan–Meier curves of OS for patients with different MPGM risk and historical differential grade groups.


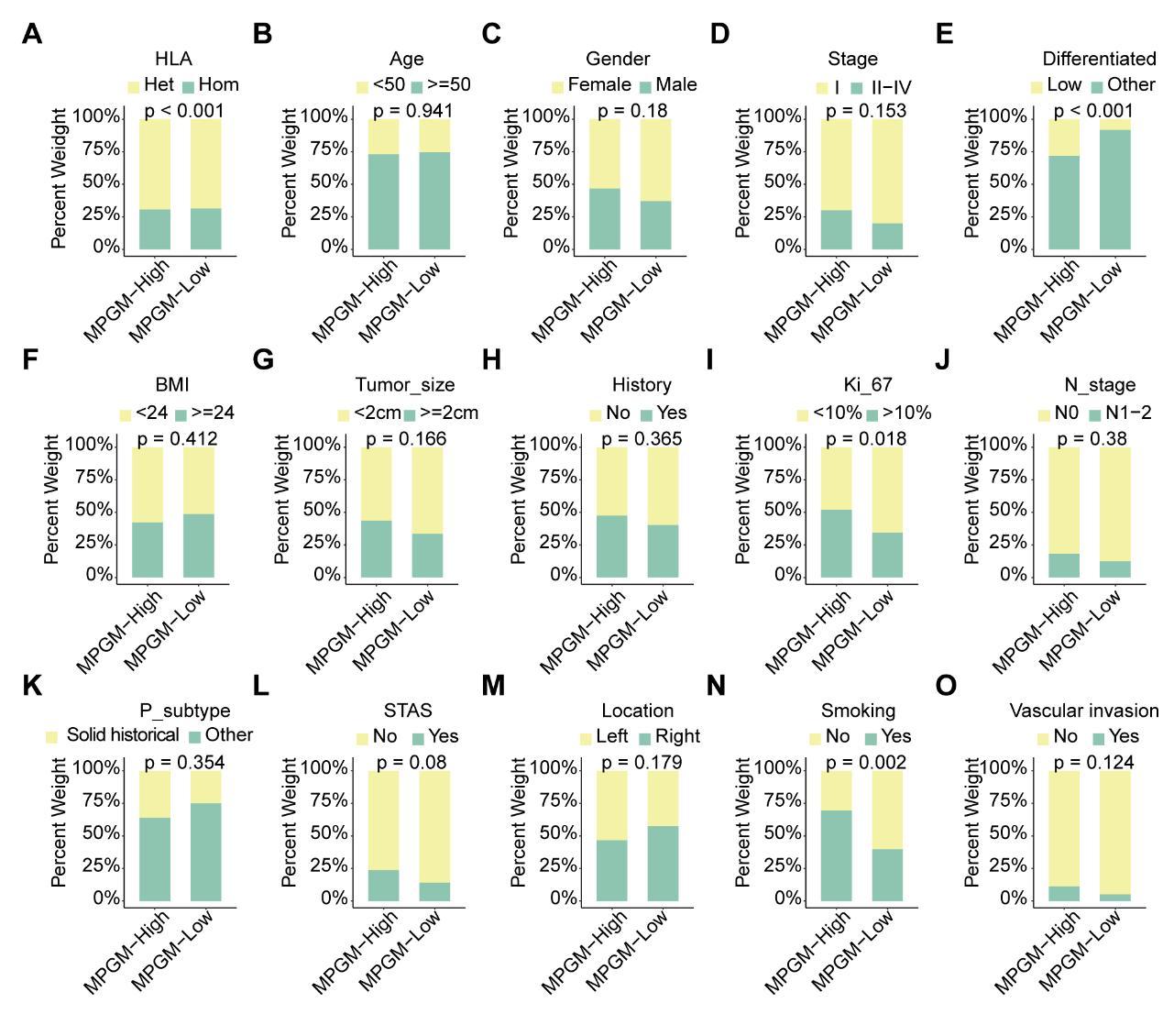


**Figure S4. Correlation analysis of MPGM model in DPH cohort.** (A) HLA variability analysis of MPGM-High and MPGM-Low groups in DPH cohort. (B-O) Proportion differences of various clinical risk factors between the MPGM-High and MPGM-Low groups.

Het: heterozygous; Hom: homozygous.

**
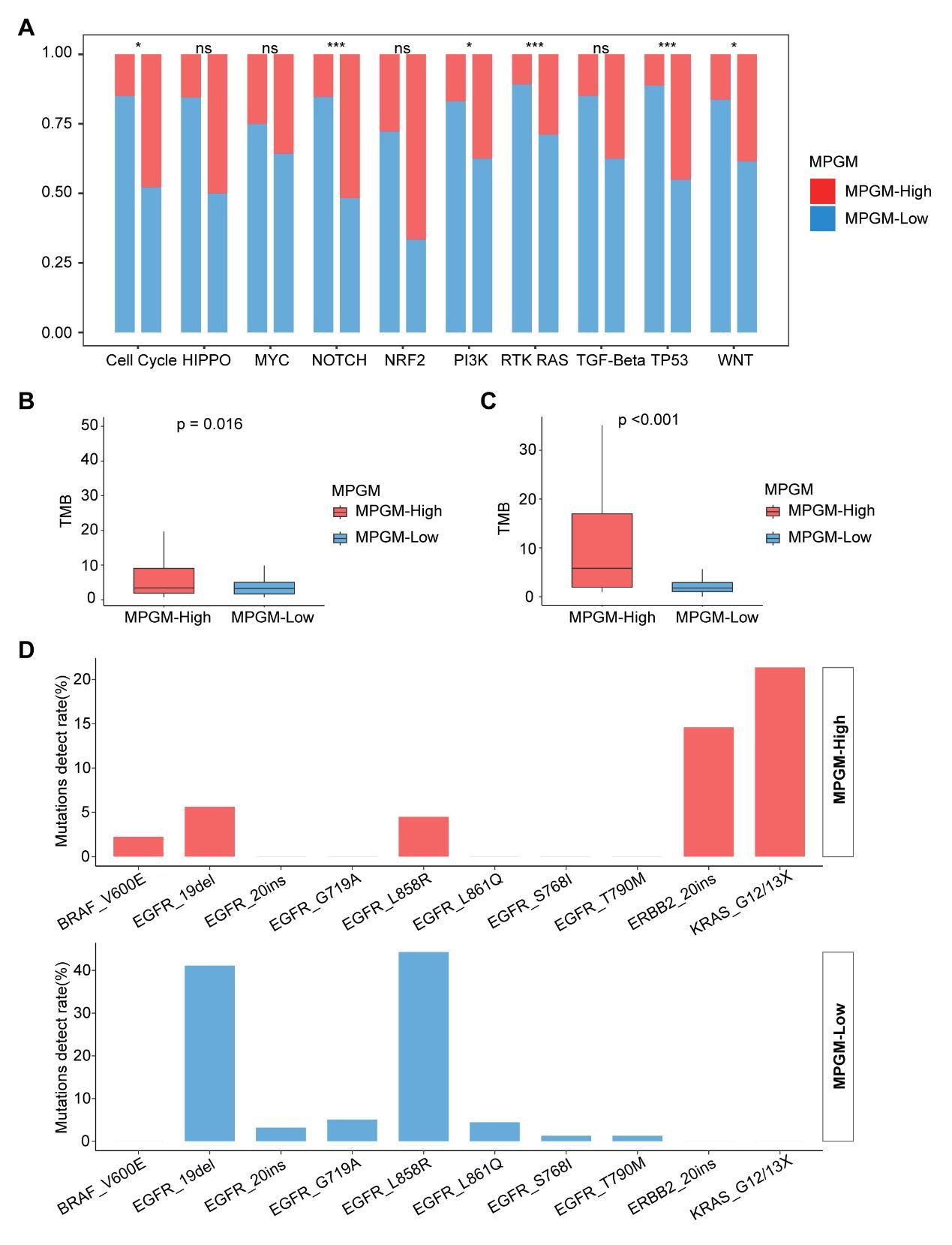
**

**Figure S5. Comparison of somatic alterations difference between DPH and EAS cohorts based on the MPGM risk model.** Comparison of the alteration frequency of genes in ten oncogenic pathways between MPGM-High and MPGM-Low groups in the EAS cohort (left) and DPH cohort (right). Comparison of TMB level between MPHGM-High and MPGM-Low groups in DPH cohort (B) and EAS cohort (C). (D) The mutation detection rate of targetable gene mutation sites between MPGM-High and MPGM-Low groups in the DPH cohort.


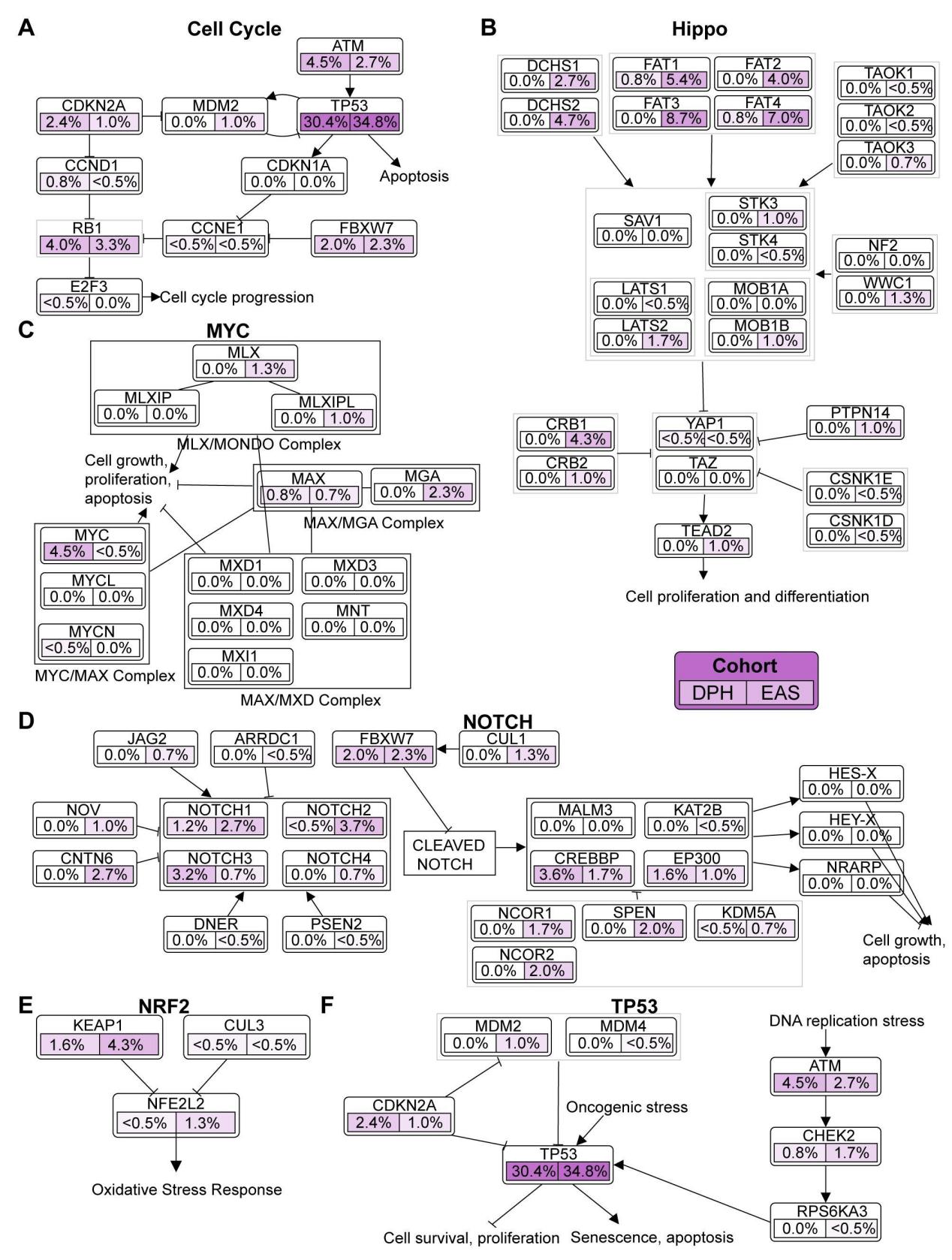


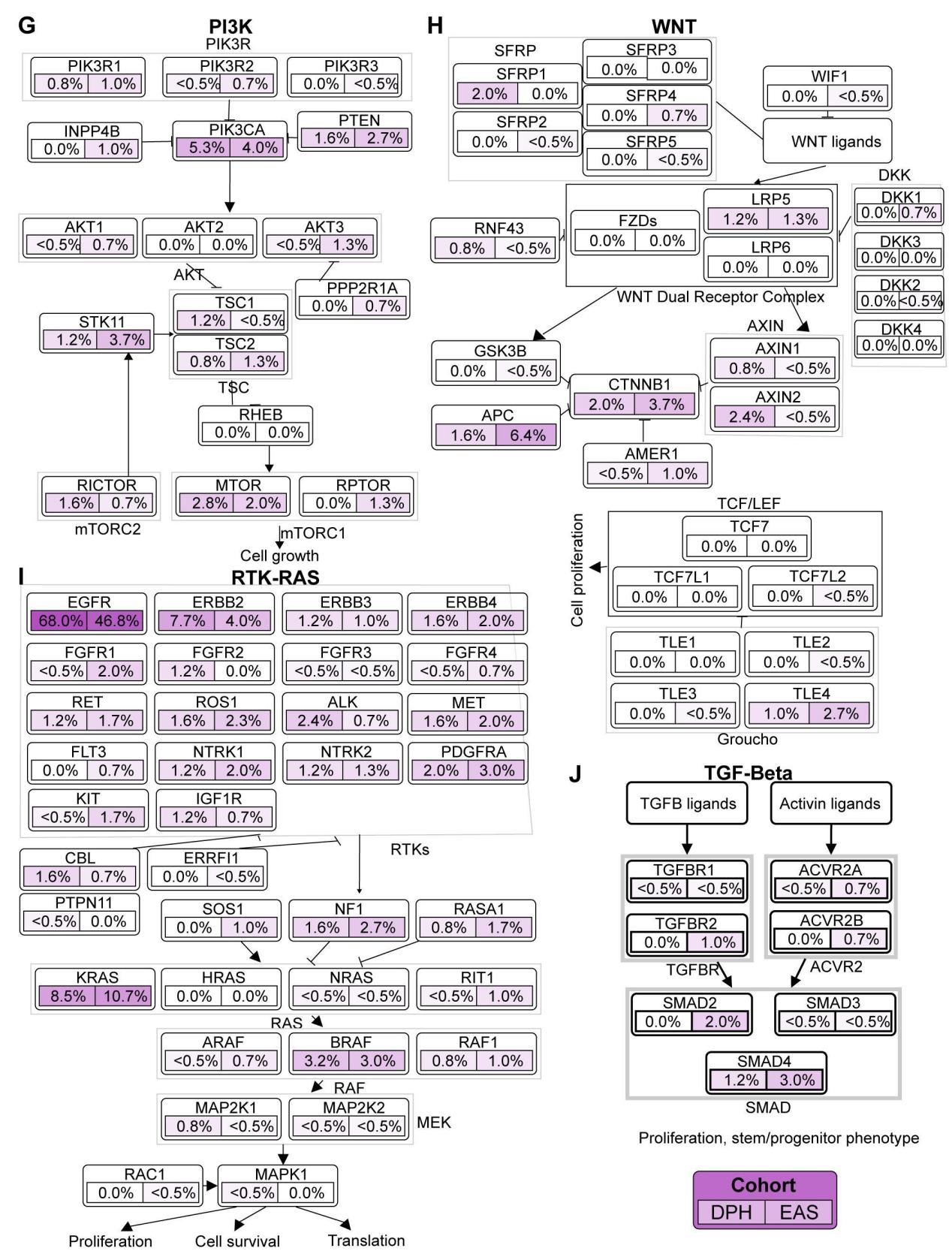


**Figure S6. Gene alterations in the ten oncogenic signaling pathways.** (A) Cell Cycle, (B) HIPPO, (C) MYC, (D) NOTCH, (E) NRF2, (F) TP53 pathways, (G) PI3K, (H) WNT, (I) RTK-RAS, and (J) TGF-Beta pathways. Numbers in the box indicate the mutation frequency of nonsynonymous somatic mutations. The left label represents the frequency in the DPH cohort, and the correct label represents the frequency in the EAS cohort.


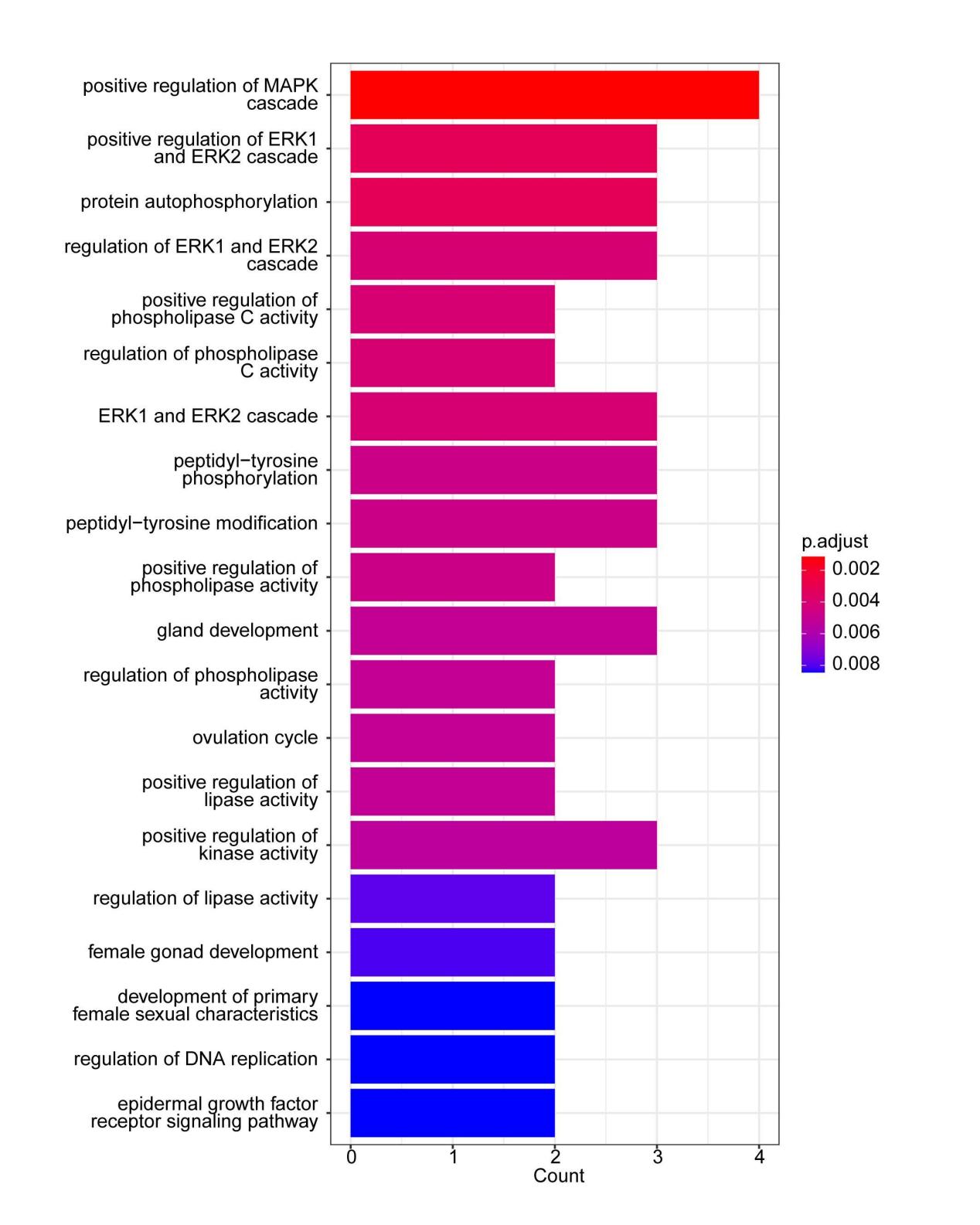


**Figure S7. Gene Ontology (GO) enrichment analysis of five model genes.**

**
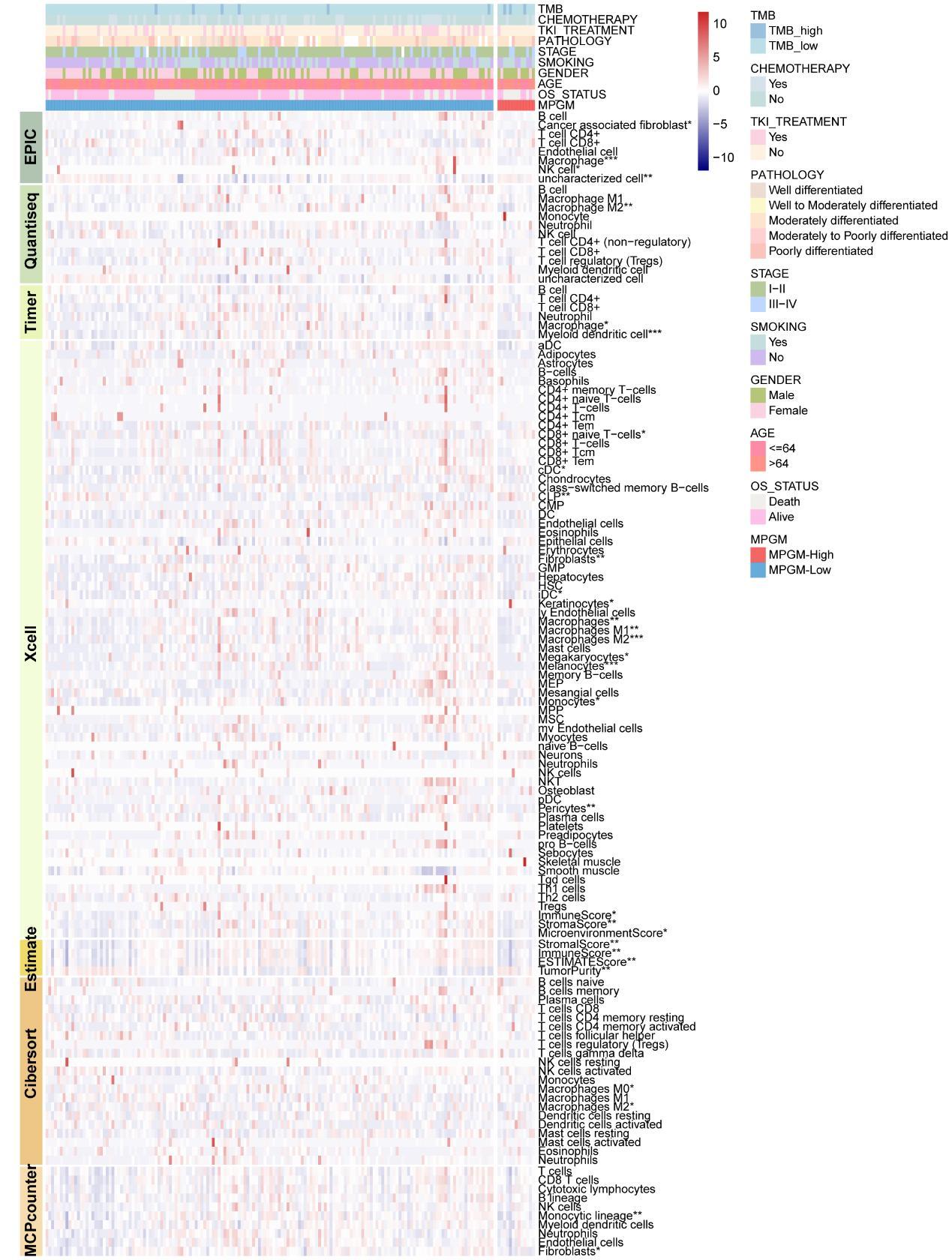
**

**Figure S8. The heatmap showed the immune cell infiltrating difference between MPGM-High and MPGM-Low groups using different immune infiltration analysis methods.**
